# Unveiling the inflammatory link: integrated analysis identifies BMP7 and PPBP as key genes in obstructive sleep apnea-related atrial fibrillation

**DOI:** 10.3389/fcell.2026.1914540

**Published:** 2026-08-20

**Authors:** Feng Li, Xing Xu, Yi-Qiu Hu, Cheng-Peng Zhang, Yi-Wei Shen, Chi Geng, Jia-Peng Chu, Hui-Yu Bai, Xiao-Song Gu, Hui Li

**Affiliations:** Department of Cardiology, The Second Affiliated Hospital of Soochow University, Suzhou, Jiangsu, China

**Keywords:** atrial fibrillation, obstructive sleep apnea, secretory proteins, immune cell infiltration, clinical cohort, rat model

## Abstract

**Background:**

Obstructive sleep apnea (OSA) is an independently modifiable risk factor for atrial fibrillation (AF). However, the molecular mechanism of OSA-related AF is elusive. This study aims to identify the key genes and pathophysiological changes underlying OSA-related AF.

**Methods:**

Three AF expression datasets, along with one OSA-related PBMC dataset and one OSA-related adipose tissue dataset, were acquired from the GEO database. DEG analysis and WGCNA were conducted to identify key AF-associated genes and OSA-related secretory proteins. PPI network analysis, functional enrichment, and cMAP were employed to uncover pathogenic genes, explore underlying mechanisms, and predict potential therapeutic compounds for OSA-related AF. LASSO regression and random forest were applied to screen candidate biomarkers and develop a diagnostic nomogram for OSA-related AF prediction. Immune cell infiltration in AF was assessed using the CIBERSORT algorithm. Finally, findings from bioinformatic analyses were validated through clinical cohort studies and animal model experiments.

**Results:**

The integrated AF dataset identified 123 AF key genes by intersecting differential expression and WGCNA. A total of 412 OSA-associated secretory proteins were screened by differential expression analysis of OSA-PBMC/adipose tissue datasets. PPI analysis identified two key modules containing 71 nodes, regarded as OSA-related AF pathogenic genes, which were mostly enriched in inflammatory and immune regulation by enrichment analysis. The cMAP analysis identified GW-9662 as a potential drug for OSA-related AF treatment. Six genes were overlapped between AF key genes and OSA-associated secretory proteins, and two hub genes were chosen as candidate biomarkers for developing nomogram with ideal diagnostic performance through machine learning. Immune cell infiltration results uncovered immune dysregulation in AF, and BMP7/PPBP were significantly associated with infiltrating immune cells. Finally, clinical and rat model validation confirmed that PPBP was upregulated and BMP7 downregulated in OSA with AF. A nomogram incorporating these genes showed good diagnostic performance (AUC = 0.843), and OSA rats exhibited increased AF susceptibility, fibrosis, and inflammation.

**Conclusion:**

This study suggests that the downregulated BMP7 and upregulated PPBP may serve as key molecular features in the pathogenesis of OSA-related AF, highlighting a shared pathological pathway between OSA and AF.

## 1 Introduction

Atrial fibrillation (AF) is the most common persistent arrhythmia in clinical practice, significantly increasing the risk of mortality, stroke, heart failure, cognitive decline, and dementia, while profoundly impacting patients’ quality of life (Svennberg et al., 2026; Rafaqat et al., 2024a). Obstructive sleep apnea (OSA) is a common sleep-related respiratory disorder. According to apnea-hypopnea index (AHI), OSA is classified as mild (5 ≤ AHI <15), moderate (15 ≤ AHI <30), or severe (AHI ≥30). OSA can induce various pathophysiological changes, including hypoxemia, autonomic dysfunction, sleep disturbances, increased chest pressure, and hypercapnia, serving as a modifiable risk factor for the initiation and progression of AF (Coso et al., 2024; Deshmukh et al., 2025). However, the causal relationship between OSA and AF remains controversial. Recent multivariable Mendelian randomization studies have shown that the association between OSA and AF may be substantially confounded by obesity, with the direct causal effect attenuated after body mass index (BMI) adjustment (Li Y. et al., 2023). This ongoing controversy underscores the need to investigate the molecular pathways underlying OSA-AF comorbidity that may operate independently of metabolic confounders.

Currently, the management of OSA in patients with concomitant AF presents significant clinical challenges. Our team previous meta-analysis (registration number CRD42023398588) indicated that continuous positive airway pressure therapy significantly reduced AF recurrence after catheter ablation [RR, risk ratio = 0.58, 95% confidence interval (CI) 0.49–0.69, *P* < 0.05] (Li et al., 2023b). However, this conclusion remains controversial due to the limited number of studies included, potential biases, and confounding factors. To address these uncertainties, our team initiated a prospective multicenter cohort study (NCT06542263 and ChiCTR2400087466) aimed at identifying optimal management strategies for this patient population. Concurrently, accumulating evidence suggests that the therapeutic variability observed in OSA-AF patients is largely attributable to the elusive molecular mechanisms and pathophysiology underlying their comorbidity (Pintilie et al., 2025). Given the confounding role of obesity in the OSA-AF association, identifying molecular pathways that are independent of BMI is of particular importance for developing targeted therapeutic strategies. Therefore, in parallel with our clinical investigation, we are pursuing mechanistic studies to screen for co-expressed genes in AF and OSA, with the goal of advancing our understanding of the pathogenic basis of their coexistence.

**FIGURE 1:**
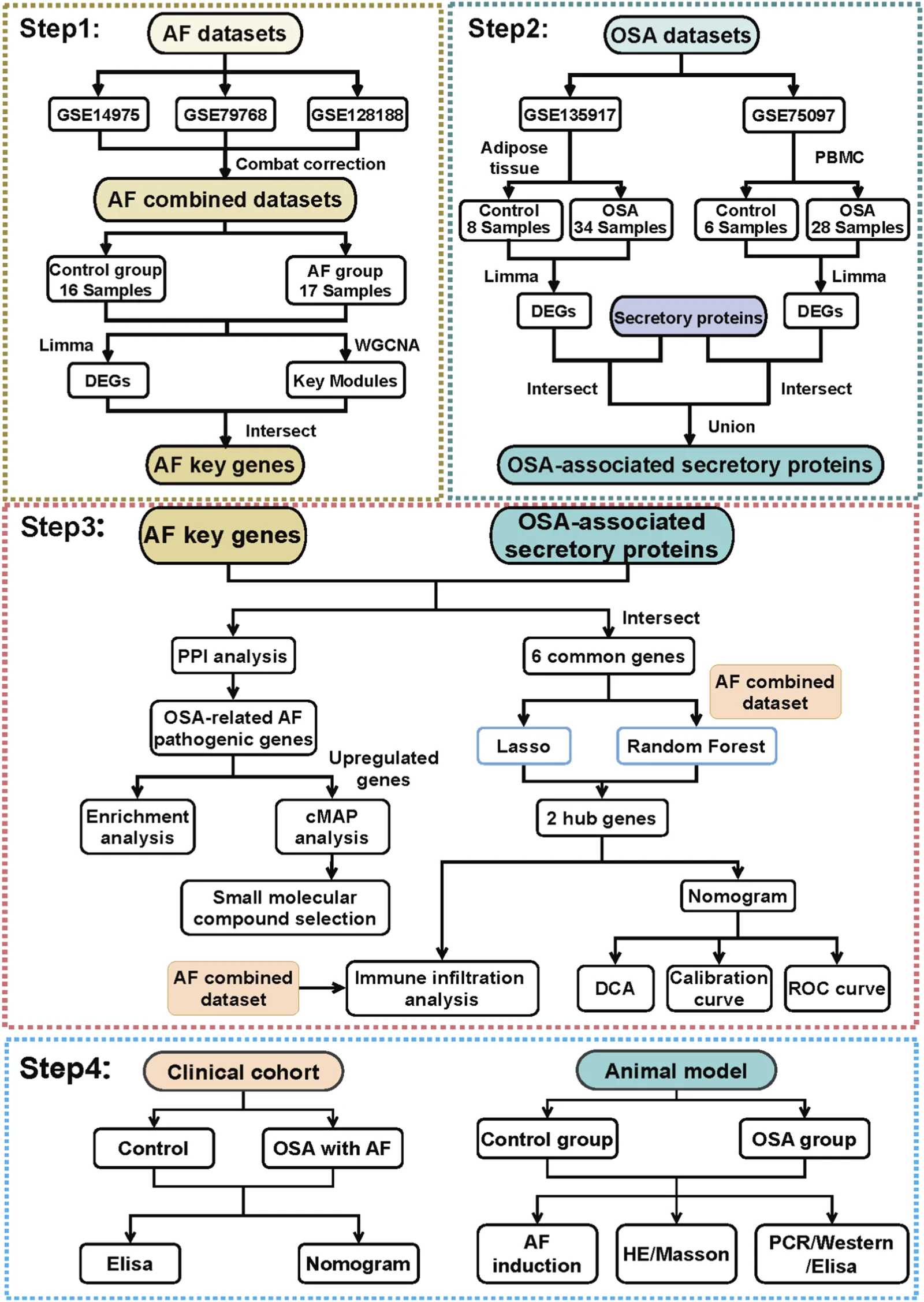
Study flowchart.

In this study, we employed an integrated approach to investigate the molecular mechanisms underlying AF with OSA (Figure 1). Gene expression datasets related to AF and OSA were obtained from the Gene Expression Omnibus (GEO) database and analyzed to identify hub genes and potential mechanisms associated with OSA-related AF pathogenesis. Also, potential compounds with therapeutic efficiency in OSA-related AF were identified via connectivity map (cMAP) analysis. Two key genes, BMP7 and PPBP, were subsequently used to develop a machine learning based diagnostic nomogram. Immune infiltration analysis was conducted to explore the relationship between these hub genes and the immune microenvironment in AF, offering mechanistic insights into the comorbidity of OSA and AF. Furthermore, their expression patterns were validated in both clinical samples and experimental OSA animal model, and the diagnostic performance of the nomogram was evaluated.

## 2 Methods

### 2.1 Microarray data preparation

Three AF datasets (GSE14975, GSE79768, and GSE128188), one OSA-related peripheral blood mononuclear cell (PBMC) dataset (GSE75097), and one OSA-related adipose tissue dataset (GSE135917) were downloaded from the GEO database (https://www.ncbi.nlm.nih.gov/geo/). Detailed information of the datasets was presented in Table 1. Based on the corresponding GPL platform annotation files (GPL570, GPL18573, GPL6244, and GPL10904), probe IDs were converted to standard human gene symbols. For cases where multiple probes corresponded to the same gene, the median expression value across samples was taken as the expression level of that gene. Gene expression data without normalization were conducted to Log2 transformation. In R (version 4.4.2), the ‘sva’ package (version 3.58.0) was used to perform batch correction on the three AF datasets.

**TABLE 1:** Datasets information for GEO.

GEO accession	Platform	Tissue source	Species	Sample size	
				SR	AF
GSE14975	GPL570	LA	*Homo sapiens*	5	5
GSE79768	GPL570	LA	*Homo sapiens*	6	7
GSE128188	GPL18573	LAA	*Homo sapiens*	5	5

GEO accession	Platform	Tissue source	Species	Sample size	
				Control	OSA
GSE135917	GPL6244	Adipose	*Homo sapiens*	8	34
GSE75097	GPL10904	PBMC	*Homo sapiens*	6	28

Abbreviation: GEO, gene expression omnibus; SR, sinus rhythm; AF, atrial fibrillation; LA, left atrium; LAA, left atrial appendage; OSA, obstructive sleep apnea; PBMC, peripheral blood mononuclear cell.

### 2.2 Identification of differentially expressed genes (DEGs)

DEGs in the AF and OSA datasets were identified using the ‘limma’ package (version 3.66.0) in R (version 4.4.2). Genes with a fold change (FC) ≥ 1.5 (log_2_FC ≥ 0.585) and *P* < 0.05 were considered significantly upregulated DEGs, while genes with log_2_FC ≤ −0.585 and *P* < 0.05 were considered significantly downregulated DEGs. Subsequently, DEGs were visualized as volcano plots and heatmaps using the ‘ggplot2’ (version 3.4.2) and ‘pheatmap’ packages (version 1.0.13), respectively.

### 2.3 Weighted gene co-expression network analysis (WGCNA) and identification of AF key genes

WGCNA was employed to identify gene modules composed of highly correlated genes, linking these modules to sample traits via module eigengenes or hub genes to elucidate biological significance and potential associations within the gene expression data (Langfelder and Horvath, 2008). The analysis proceeded as follows. First, the median absolute deviation (MAD) for each gene in the integrated AF dataset was calculated, and genes with a MAD of 0 were removed. Second, the goodSamplesGenes function in the ‘WGCNA’ package (version 1.74) in R (version 4.4.2) was used to check for outlying genes and samples. Third, the optimal soft-thresholding power (β = 6) was selected, and a scale-free co-expression gene network was constructed using the one-step network construction function in the ‘WGCNA’ package (version 1.74). Fourth, module eigengenes were obtained, and modules were correlated with traits by calculating the correlation between each module and each trait. Fifth, the modules showing the most significant positive or negative module-trait relationships were selected. Subsequently, Module Membership (MM) and Gene Significance (GS) scores were evaluated to assess module significance. AF key genes were identified by overlapping DEGs and the key modules for AF datasets, which were presented via Venn plot.

### 2.4 Acquisition of secretory protein genes and identification of OSA-associated secretory proteins

Secretory protein genes were downloaded from The Human Protein Atlas (HPA, Version 24.0) (https://www.proteinatlas.org/). This database includes genes encoding secretory proteins based on a whole-proteome scan using five signal peptide prediction methods: SignalP6.0 (https://services.healthtech.dtu.dk/services/SignalP-6.0/), Phobius (https://phobius.sbc.su.se/), SPOCTOPUS (https://topcons.net/), DeepTMHMM (https://dtu.biolib.com/DeepTMHMM/), and DeepSig (https://busca.biocomp.unibo.it/deepsig/). Genes predicted as secretory by any of these methods were included, resulting in a list of 5,119 genes.

Two sets of DEGs derived from OSA-related PBMC and adipose tissue were individually intersected with secretory protein genes, and the resulting gene sets were subsequently combined through union analysis to identify secreted protein genes associated with OSA.

### 2.5 Construction of protein-protein interaction (PPI) network and enrichment analysis

To investigate the interactions between OSA-related secretory protein-coding genes and AF-related key genes, a PPI network with significant relevance to OSA and AF was constructed using the STRING database (version 12.0), employing a confidence threshold of 0.4. The generated network was subsequently visualized in Cytoscape (version 3.10.2). Key functional modules within the PPI network were identified via the MCODE plugin (version 2.0.3) in Cytoscape (version 3.10.2), from which the two highest-scoring modules were selected for downstream investigation.

For functional characterization of OSA-related AF pathogenesis genes, Gene Ontology (GO) and Kyoto Encyclopedia of Genes and Genomes (KEGG) pathway enrichment analyses were carried out through the DAVID database (version 2025), applying a significance cutoff of *P* < 0.05. Enrichment outcomes were graphically represented using bubble plots and circus diagrams. Additionally, Gene Set Enrichment Analysis (GSEA) was performed on AF gene datasets with the ‘clusterProfiler’ package (version 4.8.1), referencing KEGG pathway gene sets (Subramanian et al., 2005). Results were ordered according to normalized enrichment score (NES) and visualized using the ‘enrichplot’ (version 1.20.0) and ‘ggplot2’ (version 3.4.2) packages.

### 2.6 CMAP analysis

The cMAP (https://clue.io) is a gene expression profile database built upon chemically induced transcriptional signatures, which enables the identification of associations among genes, diseases, and small-molecule compounds (Yang et al., 2013). By comparing our gene signature with these reference profiles, we normalized connectivity score (norm_cs), where a negative score indicates that a compound may reverse the disease-associated gene expression pattern, suggesting a potential therapeutic effect. In this study, upregulated OSA-related AF pathogenesis genes were analyzed within the cMAP platform to discover potential small-molecule therapeutics for OSA-related AF. Based on negative enrichment scores, the top ten compounds with the lowest and highest scores were selected for further evaluation.

### 2.7 Machine learning

To further prioritize candidate genes, a two-stage machine learning feature selection strategy (LASSO regression and Random Forest) was applied. Briefly, LASSO regression with 10-fold cross-validation was performed using the ‘glmnet’ package (version 4.1.10), followed by Random Forest algorithm using the ‘randomForest’ package (version 4.7.1.2) to rank gene importance. The overlapping genes selected by both methods were considered as prioritized candidates. To confirm that the selected genes encode experimentally validated secretory proteins, hub genes were further screened using the GeneCards database (https://www.genecards.org/, version 5.25) based on the presence of experimental evidence for “Secreted” classification in their subcellular localization annotation. Detailed parameter settings are described in the Supplementary Methods.

### 2.8 Nomogram construction and diagnostic marker prediction model assessment

Forest plots for PPBP and BMP7 were generated using the ‘ggplot2’ package (version 3.4.2) to visualize odds ratios (OR) with 95% CI from the logistic regression models. The diagnostic nomogram was constructed based on the two hub genes (BMP7 and PPBP) using the ‘rms’ package (version 8.1.1). To evaluate the discriminative capability of each hub gene and the integrated nomogram in classifying OSA-related AF, receiver operating characteristic (ROC) curves were generated, and corresponding areas under the curve (AUC) were computed. ROC analysis was further performed to examine the performance of the nomogram in distinguishing between OSA-related AF subtypes. Finally, the predictive accuracy and clinical net benefit of the nomogram were assessed using calibration curves and decision curve analysis (DCA), respectively.

### 2.9 Immune infiltration analysis

Immune cell infiltration in AF was evaluated by applying the CIBERSORT algorithm (version 0.1.0) to gene expression profiles. Bar plots generated using the ‘ggplot2’ package (version 3.4.2) were employed to visualize the relative abundance of infiltrating immune cells across samples. Differences in the proportions of 22 immune cell types between OSA-related AF and control groups were analyzed with the Wilcoxon test (*P* < 0.05 considered significant) and illustrated via a stacked bar chart using the ‘ggplot2’ package (version 3.4.2). Interrelationships among these immune cell types were further depicted using the ‘corrplot’ package (version 0.95). Finally, associations between diagnostic biomarker expression and immune cell infiltration were assessed through Spearman’s rank correlation analysis, with statistical significance set at *P* < 0.05.

### 2.10 Patient samples collection

This study included control individuals (no/mild OSA without AF, n = 40) and OSA with AF individuals (moderate-to-severe OSA with concomitant AF, n = 20) who visited the Department of Cardiology at Second Affiliated Hospital of Soochow University from January 2025 to December 2025. Moderate-to-severe OSA was determined via portable sleep monitoring device with AHI greater than 15/hour; Otherwise, it would be classified as no/mild OSA. AF includes persistent AF and paroxysmal AF. Moreover, serum samples from control individuals and OSA with AF individuals were also collected from Second Affiliated Hospital of Soochow University. Patients with neuromuscular disorders, pulmonary hypopnea, severe lung diseases, secondary atrial fibrillation, as well as history of myocardial infarction, percutaneous coronary intervention, and cardiac surgery in the past 3 months were excluded. The patients’ baseline information was shown in Table 2. In OSA with AF patients, a total of 13 (65%) patients had paroxysmal AF, and 7 (35%) patients persistent AF. OSA with AF patients displayed a higher AHI and lower lowest oxygen saturation (LSaO2) (both *P* < 0.001). Interestingly, the OSA with AF patients seemed to have a higher BMI compared with the control group (26.03 ± 3.43 vs. 24.36 ± 3.06 kg/m^2^; *P* = 0.059). Notably, LVEF was comparable between the two groups, reflecting the inclusion of patients with similar cardiac function in our cohort. More importantly, this comparability provides an opportunity to investigate OSA-related molecular alterations that are independent of systolic dysfunction. The study protocol was approved by the Institutional Research Ethics Committee of the Second Affiliated Hospital of Soochow University (Approval No. JD-LK2024034-IR01) in accordance with the Declaration of Helsinki. Written informed consent to participate was obtained from all participants prior to sample collection.

**TABLE 2:** Patients baseline information.

Items	Control (n = 40)	OSA and AF (n = 20)	*P* Value
Age [mean (SD)]	67.62 (8.38)	70.20 (5.21)	0.214
BMI [mean (SD)]	24.36 (3.06)	26.03 (3.43)	0.059
Gender (%)	​	​	0.586
Male	18 (45.0)	11 (55.0)	​
Female	22 (55.0)	9 (45.0)	​
Smoking (%)	​	​	0.511
Yes	7 (17.5)	5 (25.0)	​
No	33 (82.5)	15 (75.0)	​
Alcohol (%)	​	​	0.718
Yes	6 (15.0)	4 (20.0)	​
No	34 (85.0)	16 (80.0)	​
Hypertension (%)	​	​	0.756
Yes	30 (75.0)	16 (80.0)	​
No	10 (25.0)	4 (20.0)	​
DM (%)	​	​	0.278
Yes	5 (12.5)	5 (25.0)	​
No	35 (87.5)	15 (75.0)	​
Stroke/TIA (%)	​	​	1.000
Yes	7 (17.5)	4 (20.0)	​
No	33 (82.5)	16 (80.0)	​
CVD (%)	​	​	1.000
Yes	9 (22.5)	4 (20.0)	​
No	31 (77.5)	16 (80.0)	​
LVEF [mean (SD)]	57.85 (10.04)	56.40 (8.89)	0.586
AHI [mean (SD)]	5.19 (3.70)	26.45 (11.03)	**<0.001**
LSaO2 [mean (SD)]	86.80 (5.91)	78.10 (10.25)	**<0.001**

Abbreviation: OSA, obstructive sleep apnea; AF, atrial fibrillation; BMI, body mass index; DM, diabetes mellitus; TIA, transient ischemic attack; CVD, cardiovascular disease; LVEF, left ventricular ejection fraction; AHI, Apnea-hypopnea index; LSaO2, lowest oxygen saturation; SD, standard deviation. The bold values indicate statistical significance.

### 2.11 Animals

Male 6–8 weeks Sprague-Dawley (SD) rats were purchased from Changzhou Cavins Laboratory Animal Co., Ltd. As previously described for the OSA rat model (Breuillard et al., 2023), after 1 week of acclimatization in an SPF facility (23 °C, 60% humidity, 12 h light/dark cycle, *ad libitum* food/water), rats were subjected to intermittent hypoxia for 8 h daily (9 AM-5 PM) over 8 weeks. Twenty rats were randomly divided into two groups, including OSA group (n = 10) and control group (n = 10). For OSA group, each intermittent hypoxia cycle lasted 60 s (including 9 s of nitrogen infusion, 16 s at 5% O_2_, and 35 s of air infusion to restore 21% O_2_). For control group, rats were maintained under normoxic conditions in independent ventilated cage. Body weight was monitored weekly. Animal experiments were approved by the Institutional Animal Care and Use Committee of Soochow University (SYXK(SU)2021–0065).

### 2.12 AF induction

In this study, rats were anesthetized with 2% isoflurane and placed on a heating pad to maintain the body temperature approximately at 37 °C (Li et al., 2023c). The rats underwent tracheal intubation and were connected to the ventilator. The ADInstruments physiological data acquisition and analysis system and the MappingLab VCS-3002 programmable stimulator were used to synchronously record surface electrocardiogram (ECG). Atrial pacing threshold was found using 2 ms pulses at a cycle length of 150 ms or 20 ms shorter than the spontaneous sinus. The AF induction was performed using transesophageal atrial pacing, with the pacing setting set at 3 times the atrial pacing threshold. Burst pacing containing 200 impulses at 50 Hz was used to induce AF, and AF was determined based on rapid and irregular f waves replace P waves and cause rapid atrial responses with a duration of ≥ 1s (Zhang et al., 2020). LabChart 8 (version 8.1.13) was used to measure electrical parameters.

### 2.13 Hematoxylin and eosin (HE) and masson staining

Upon isolation, fresh left atrial tissue from rats was processed for histological examination. For HE staining, tissue samples were fixed in 4% paraformaldehyde (Sigma, P6148), followed by dehydration through a graded ethanol series, paraffin infiltration, and embedding. Sections were cut at a thickness of 4 μm and stained using a commercial HE staining kit (Servicebio, G1005). For Masson’s trichrome staining, paraffin-embedded sections of 5 μm thickness were prepared and stained with Masson’s trichrome solution (Servicebio, G1006). Stained sections were observed and imaged under an optical microscope at ×40 magnification for qualitative assessment of tissue morphology and collagen deposition, respectively.

### 2.14 Real-time PCR analysis

Total RNA was isolated from rat atrial tissue using Trizol reagent (Invitrogen, United States) and subsequently reverse-transcribed into complementary DNA (cDNA) with a commercial kit (TAKARA, Japan). Quantitative real-time PCR was performed in 96-well plates to measure the mRNA expression levels of PPBP, BMP7, IL-1β, IL-6, and TNF-α. Relative expression was determined via the 2^−ΔΔCT^ method. The following primer sequences were used for cDNA amplification: TCC​CGT​GTG​AAT​GTG​TTC​CG (forward) and ATC​ATA​GGG​GCA​GTC​GGG​T (reverse) for PPBP; GACCCCAGAACAAGCAA (forward) and CTC​ACA​GTA​GTA​GGC​AGC​AT (reverse) for BMP7; GCA​ACT​GTT​CCT​GAA​CTC​AAC​T (forward) and ATC​TTT​TGG​GGT​CCG​TCA​ACT (reverse) for IL-1β; TAG​TCC​TTC​CTA​CCC​CAA​TTT​CC (forward) and TTG​GTC​CTT​AGC​CAC​TCC​TTC (reverse) for IL-6; CCC​TCA​CAC​TCA​GAT​CAT​CTT​CT (forward) and GCT​ACG​ACG​TGG​GCT​ACA​G (reverse) for TNF-α; CAAGTTCAACGGCACAG (forward) and CCA​GTA​GAC​TCC​ACG​ACA​T (reverse) for GAPDH.

### 2.15 Western blot analysis

Left atrial tissue samples from both OSA and control rats were homogenized using RIPA lysis buffer (Thermo Fisher Scientific, #89901) containing phosphatase and protease inhibitors (Thermo Fisher Scientific, #78442). Following protein separation by SDS-PAGE, samples were transferred onto PVDF membranes. The membranes were subsequently incubated overnight at 4 °C with primary antibodies specific for PPBP (Thermo Fisher Scientific, #PA5-115070) and BMP7 (Abcam, #ab129156). To verify consistent protein loading across lanes, the same membranes were stripped and reprobed with antibodies against GAPDH (Cell Signaling Technology, #5174) or β-actin (Cell Signaling Technology, #4970). Protein band intensities were analyzed and quantified using ImageJ software (Scion Corp., USA).

### 2.16 ELISAs

For patients, plasma samples were collected from fasting patients during admission and stored at −80 °C. Plasma PPBP levels and BMP7 were measured, according to the manufacturer’s instructions, in triplicate using a Human chemokine CXCL7/PPBP ELISA kit (Wuhan USCN Business Co., Ltd., USEA370Hu) and Human BMP-7 Quantikine ELISA Kit (USA R&D Systems, Inc., DBP700).

For animals, 8 weeks after model induction, the rats were euthanized, and blood samples were collected via the inferior vena cava. Plasma PPBP levels and BMP7 were detected based on manufacturer’s instructions using Rat chemokine CXCL7/PPBP ELISA kit (Wuhan USCN Business Co., Ltd., USEA370Ra) and Rat BMP7 ELISA kit (Wuhan USCN Business Co., Ltd., USEA799Ra). Moreover, multiple inflammatory factors were detected from tissue level. Frozen rat atrial tissues were initially processed in cold PBS. After homogenization and centrifugation (4 °C, 3,000 rpm, 10 min), the levels of IL-1β, IL-6, and TNF-α in the rat atrial tissues were strictly determined according to the requirements of the ELISA kit from Sigma-Aldrich (Product Number: RAB0479, RAB0277, and RAB0311, respectively).

### 2.17 Statistical analysis

Statistical analyses were conducted using R software (version 4.4.2), GraphPad Prism 7, and SPSS 25.0. Continuous data were expressed as mean ± SEM. Continuous variables were performed using Student’s t-test with normally distributed variables and Kruskal–Wallis test with variables not normally distributed. Categorical variables were compared using the chi-square test or Fisher’s exact test, as appropriate. Univariate and multivariable logistic regression analyses were conducted to evaluate the associations of PPBP and BMP7 with OSA-related AF, with BMI adjustment in the multivariable models to address potential confounding. To facilitate clinical interpretation, PPBP was scaled per 100-unit increase and BMP7 per 10-unit increase. The *P* < 0.05 was considered statistically significant.

## 3 Results

### 3.1 Data processing and DEGs screening for AF

**FIGURE 2:**
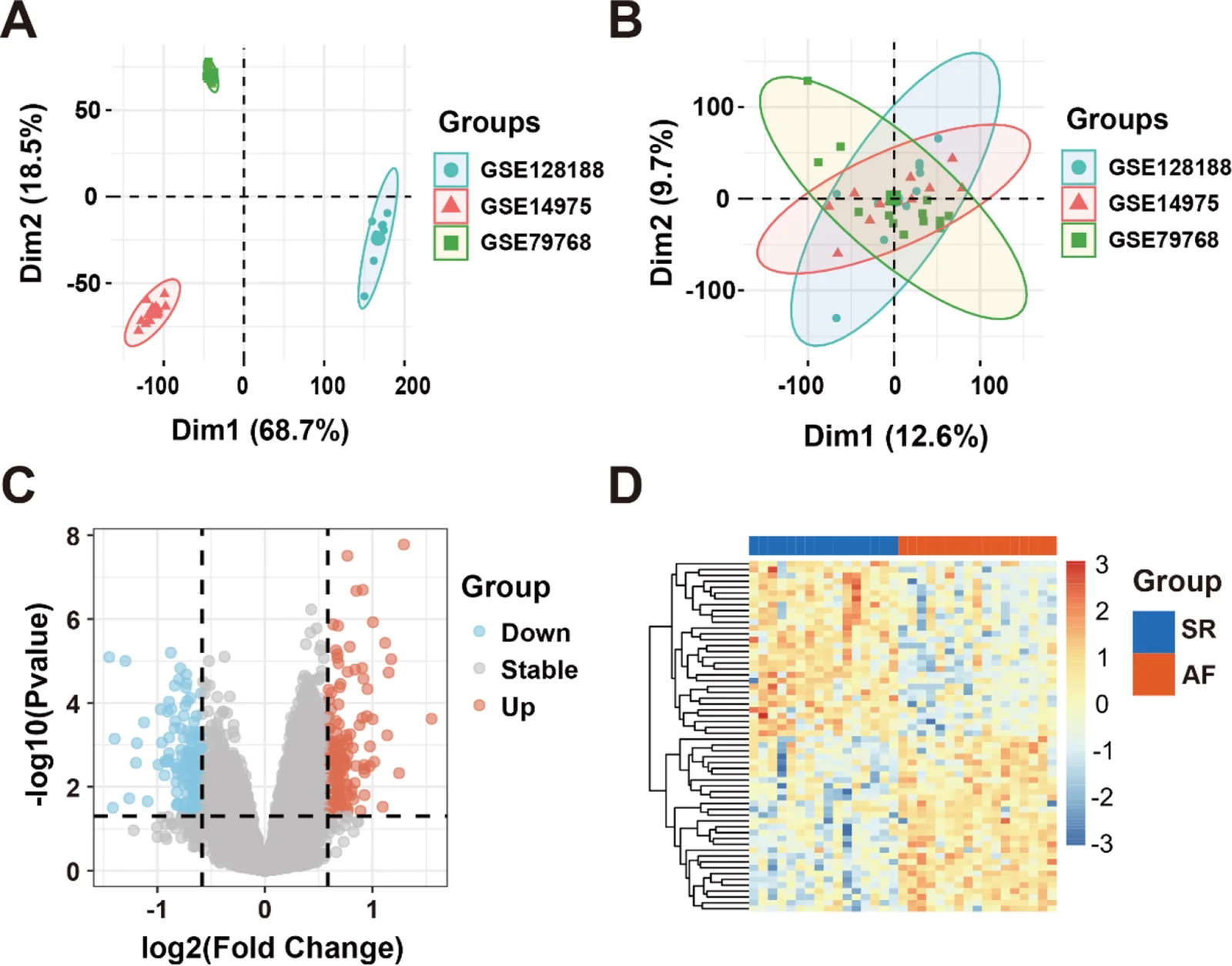
DEGs screening for AF. **(A,B)** Before and after batch correction for AF datasets; **(C,D)** Volcano plot and heatmap for AF datasets. DEGs, Differentially expressed genes; AF, Atrial fibrillation.

Three publicly available datasets containing AF and control samples were acquired from the GEO database. Following batch effect correction and integration, a combined AF dataset was established and subjected to normalization, yielding a total of 17 AF samples and 16 control samples. Post-integration analysis confirmed the absence of significant batch-related disparities across the three source datasets (Figures 2A,B). Differential expression analysis performed on the merged dataset identified 318 DEGs, comprising 168 upregulated and 150 downregulated genes, applying thresholds of *P* < 0.05 and |log_2_FC| ≥ 0.585. The resulting DEGs were graphically represented using a volcano plot and a heatmap (Figures 2C,D).

### 3.2 Construction of WGCNA and identification of key module

**FIGURE 3:**
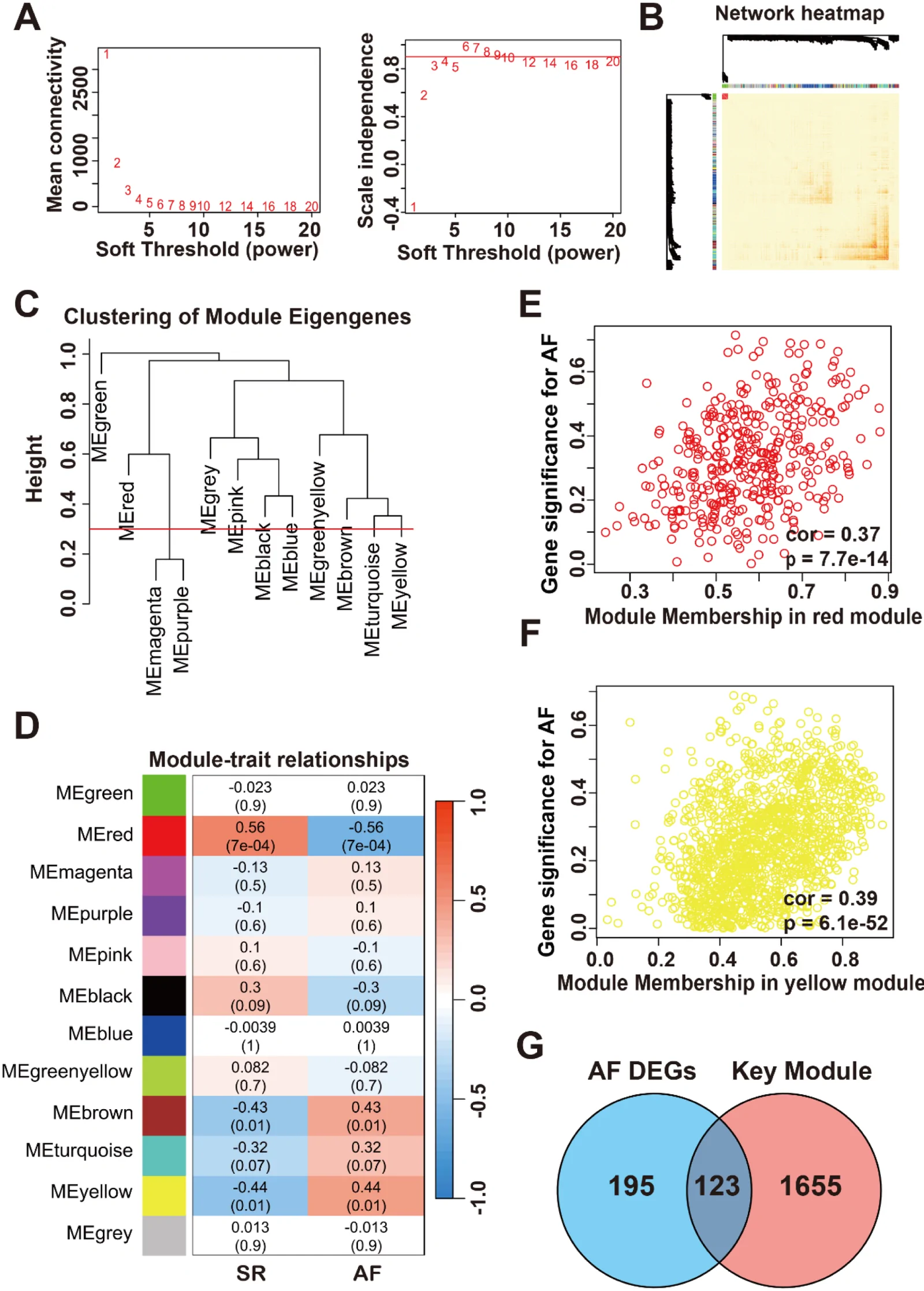
WGCNA for AF datasets. **(A)** Mean connectivity and scale independence for WGCNA construction; **(B)** Topological overlap matrix; **(C)** Hierarchical clustering of module eigengenes; **(D)** Correlation analysis between AF status and each gene module; **(E,F)** Top two modules; **(G)** AF key genes with Venn plot. AF, Atrial fibrillation.

To identify the key genes in AF, WGCNA was performed. After evaluating scale-free topology and mean connectivity, a soft-thresholding power (β) of six was chosen to build the co-expression network (Figure 3A). Using this parameter, 12 distinct modules were generated, with their topological overlap matrix (TOM) displayed in Figure 3B. The hierarchical clustering of module eigengenes is presented in Figure 3C.

Correlations between AF status and each gene module were subsequently evaluated (Figure 3D). The yellow module showed the highest positive correlation with AF (r = 0.44, *P* = 0.01) and contained 1,396 genes, whereas the red module exhibited the strongest negative correlation (r = −0.56, *P* = 7 × 10^−4^), comprising 382 genes. Based on these results, the yellow and red modules were designated as key modules for further investigation. Module membership (MM) was found to be significantly correlated with gene significance (GS) for AF in both the yellow (r = 0.39, *P* = 6.2 × 10^−52^) and red modules (r = 0.37, *P* = 7.7 × 10^−14^), as shown in Figures 3E,F. Collectively, a total of 1,778 genes from these two modules were considered significantly associated with AF.

Finally, genes from the key WGCNA modules were intersected with AF-related DEGs, resulting in a total of 123 overlapping genes (Figure 3G). These overlapping genes were defined as key candidate genes for AF and carried forward for subsequent analysis.

### 3.3 Identification of secreted related DEGs in OSA

**FIGURE 4:**
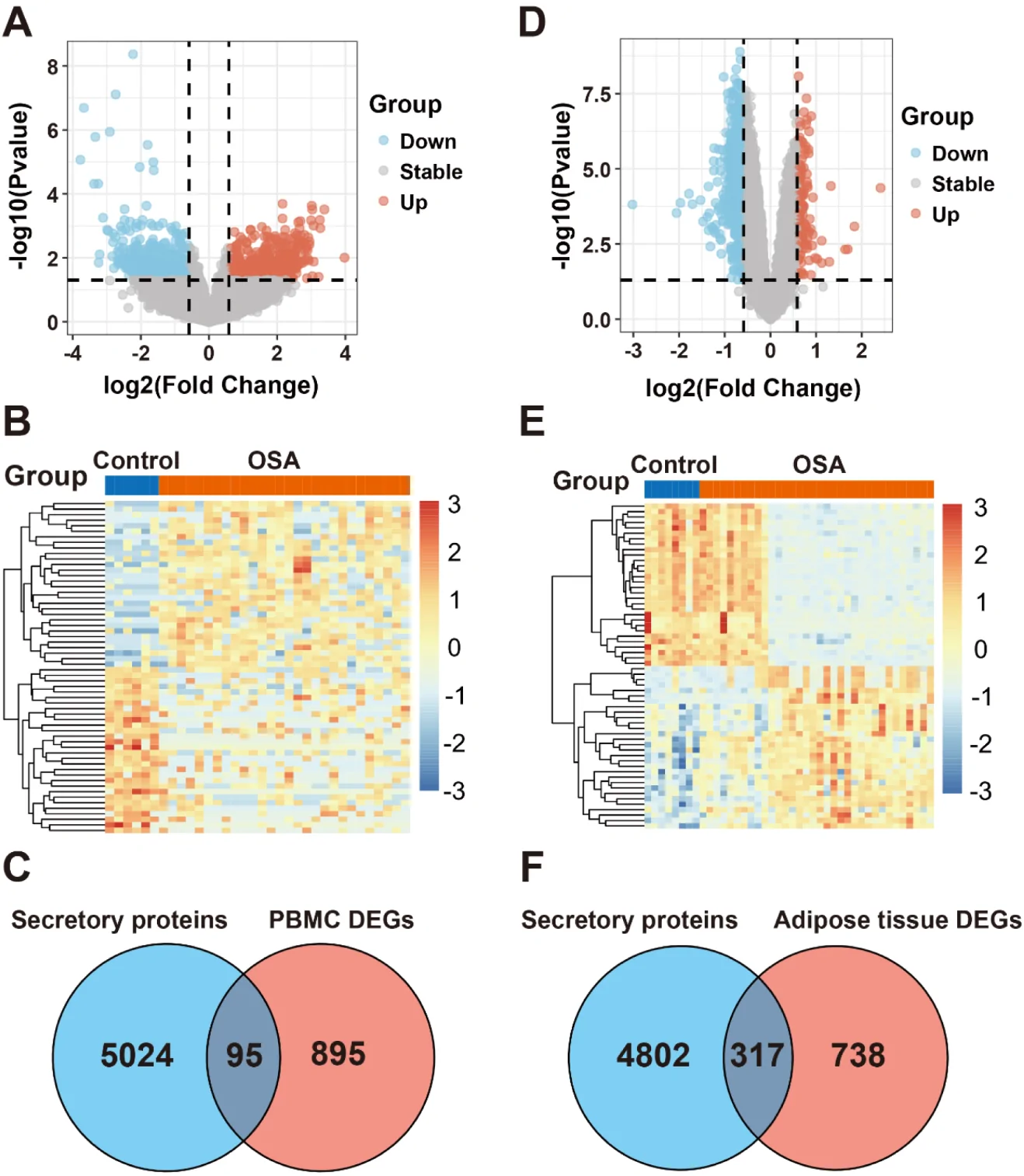
OSA DEGs screening. **(A,B)** DEGs with volcano plots and heatmaps for PBMCs; **(C)** Overlapping PBMC DEGs and secretory protein–related genes; **(D,E)** DEGs with volcano plots and heatmaps for adipose tissue; **(F)** Overlapping adipose tissue DEGs and secretory protein-related genes. OSA, Obstructive sleep apnea; DEGs, Differentially expressed genes; AF, Atrial fibrillation; PBMC, Peripheral blood mononuclear cell.

To identify pathogenic genes that may link OSA to AF, we obtained gene expression datasets from the GEO database, comprising PBMC and adipose tissue samples from individuals with OSA. Differential expression analysis was conducted separately for each dataset. In PBMCs, a total of 990 DEGs were identified, consisting of 564 upregulated and 426 downregulated genes. In adipose tissue, a total of 1,055 DEGs were detected, including 131 upregulated and 924 downregulated genes. Volcano plots and heatmaps were generated to visualize these DEGs for PBMCs (Figures 4A,B) and for adipose tissue (Figures 4D,E).

To concentrate on potential systemic signaling mediators, DEGs from each tissue were intersected with genes encoding secreted proteins. This process yielded 95 secreted-protein–related genes from PBMCs (Figure 4C) and 317 from adipose tissue (Figure 4F). Following combination of the two gene sets, a total of 412 unique OSA-associated secretory proteins were retained for subsequent investigation.

### 3.4 Protein-protein interaction network and functional enrichment of the pathogenic genes involved in OSA-related AF

**FIGURE 5:**
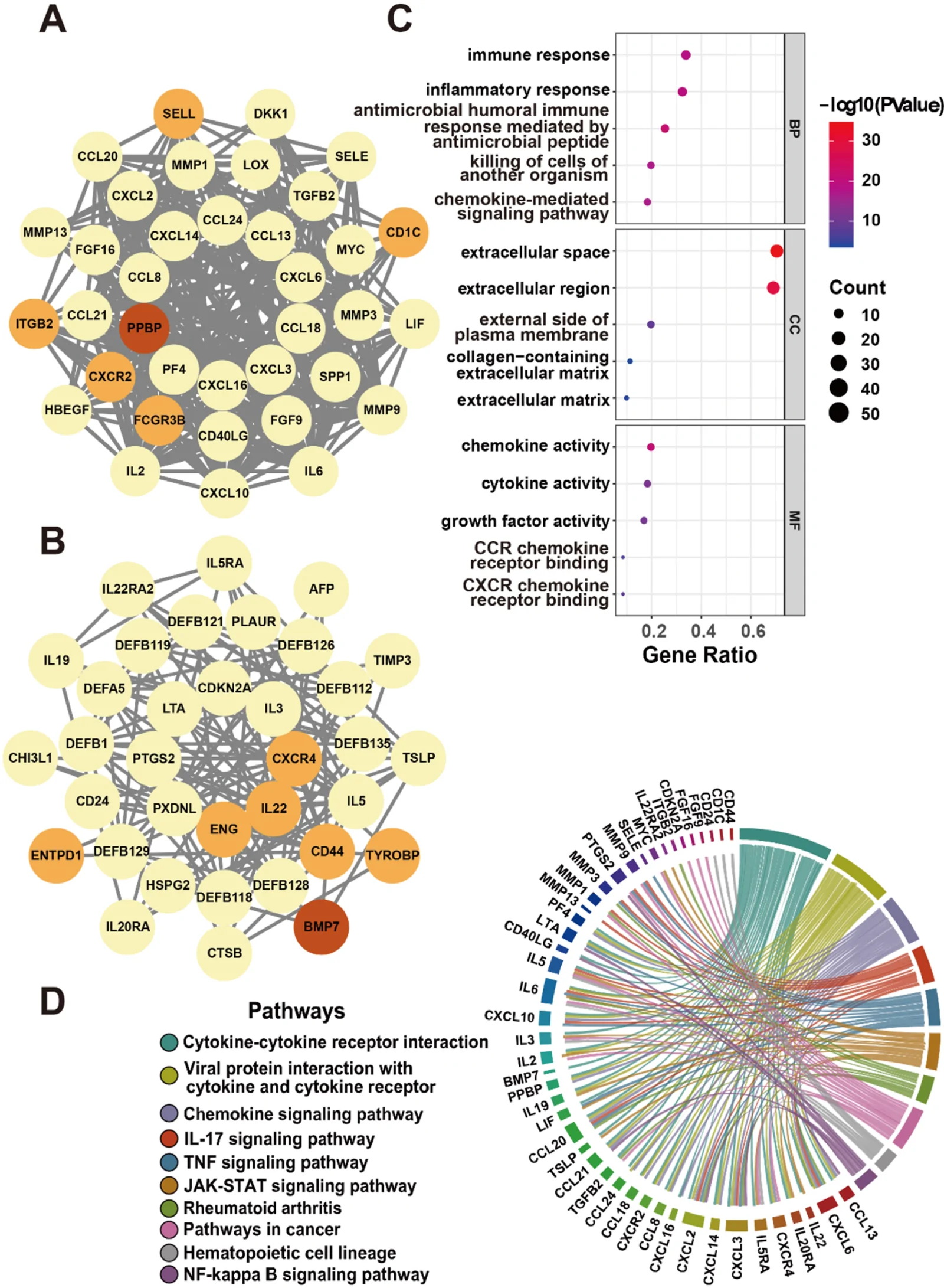
PPI construction and enrichment analysis. **(A,B)** The top two most significant modules, in which light-yellow points are derived from OSA key genes, dark-yellow points are derived from AF key genes, and brown points are common to both OSA and AF key genes; **(C,D)** GO enrichment and KEGG pathway analyses based on 71 candidate genes identified from the PPI network modules. OSA: Obstructive sleep apnea; AF, Atrial fibrillation; BP, Biological process; CC, Cellular component; MF, Molecular function.

A total of 519 candidate genes were compiled by merging AF-associated key genes with genes encoding OSA-related secretory proteins. Using Cytoscape, these candidate genes were visualized as a network, and the two most significant modules (Figures 5A,B) were identified with the MCODE plugin, comprising a total of 71 genes which were designated as pathogenic genes for OSA-related AF. To elucidate their functional roles and underlying mechanisms, these 71 pathogenic genes were subjected to GO functional enrichment and KEGG pathway analyses via the DAVID database. In the GO biological process (BP) category, genes were predominantly enriched in immune response, inflammatory response, and chemokine-mediated signaling. Cellular component (CC) analysis indicated primary localization in the extracellular space, extracellular region, and extracellular matrix. Molecular function (MF) results highlighted enrichment in chemokine activity, cytokine activity, and CXCR chemokine receptor binding (Figure 5C). KEGG pathway analysis further revealed significant associations with cytokine-cytokine receptor interaction, chemokine signaling pathway, TNF signaling pathway, and NF-κB signaling pathway (Figure 5D).

**FIGURE 6:**
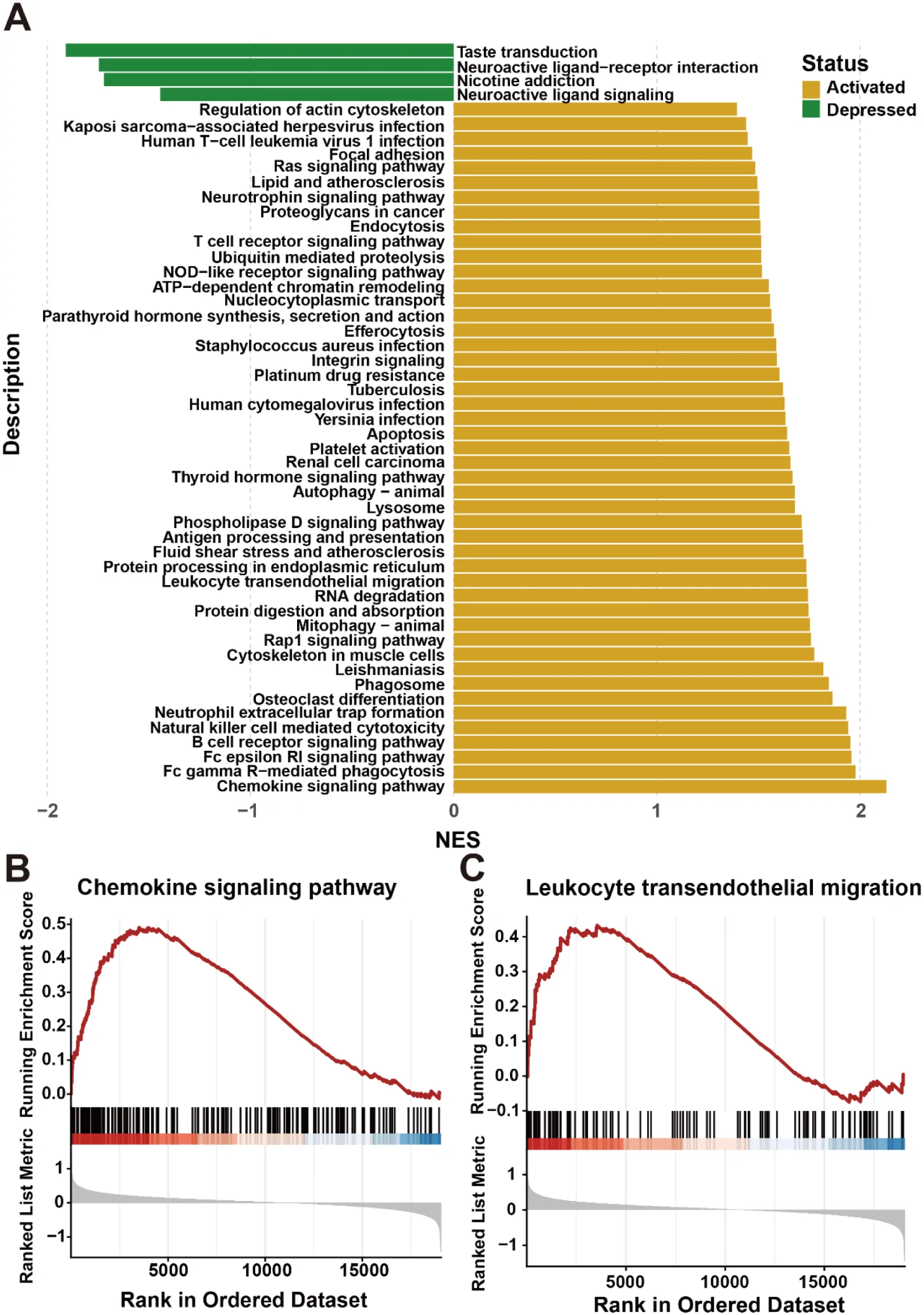
GSEA analysis. **(A)** GSEA results including depressed and activated pathway; **(B,C)** Chemokine signaling pathway and leukocyte transendothelial migration.

Additionally, GSEA was conducted using all AF-related DEGs without applying filtering thresholds. The results demonstrated significant upregulation of pathways including chemokine signaling, Fc gamma R-mediated phagocytosis, Fc epsilon RI signaling pathway, B Cell receptor signaling, and leukocyte transendothelial migration (Figures 6A–C). Conversely, pathways such as taste transduction, neuroactive ligand-receptor interaction, nicotine addiction, and neuroactive ligand signaling were markedly downregulated (Figure 6A).

### 3.5 Identification of candidate small-molecular compounds for AF treatment

**FIGURE 7:**
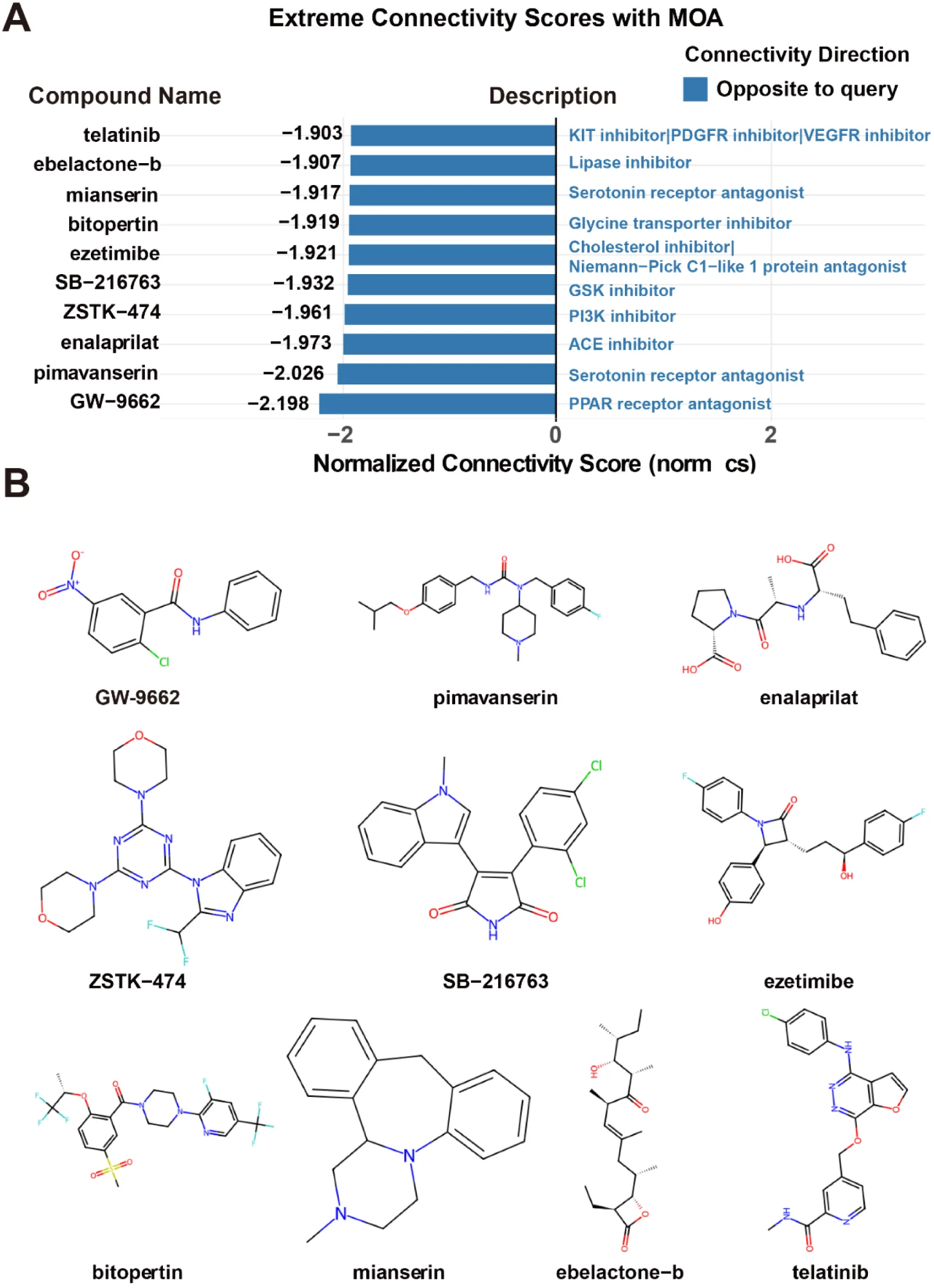
cMAP analysis. **(A)** The top ten candidate small-molecular compounds with drug MOA; **(B)** Chemical structures of ten candidate small-molecular compounds. MOA, Mechanism of action.

To identify potential therapeutic agents for OSA-related AF, a total of 92 upregulated OSA-related AF pathogenic genes were analyzed using the cMAP database to identify small-molecule compounds capable of reversing their dysregulated expression patterns. Based on negative enrichment scores, the top ten compounds predicted to most effectively counteract these gene expression changes were identified. These candidate compounds, including GW-9662, pimavanserin, enalaprilat, ZSTK-474, SB-216763, ezetimibe, bitopertin, mianserin, ebelactone-b, and telatinib, represent potential pharmacological agents for OSA-related AF treatment. Their corresponding biological pathways and chemical structures are presented in Figures 7A,B, respectively.

### 3.6 Screening of hub genes harboring diagnostic value via machine learning and construction of a diagnostic model in OSA-related AF

Given the potential importance of secretory proteins differentially expressed in both AF and OSA for OSA-related AF pathogenesis, we identified six overlapping genes between OSA-associated secretory proteins and AF-related key genes (Supplementary Figure S1A). To prioritize the most robust candidates from these six genes, four genes were identified based on LASSO regression and Random Forest (Supplementary Figure S1B-E). Violin plots depicting expression levels of these four genes in the AF dataset and the OSA-related adipose tissue dataset are shown in Supplementary Figure S2. Among them, two genes (NPY1R and PPBP) were upregulated, and two genes (BMP7 and METTL7B) were downregulated in AF and OSA, respectively, compared to control. Of these, only PPBP and BMP7 were confirmed to encode experimentally verified secreted proteins, which were determined as hub genes for OSA-related AF.

**FIGURE 8:**
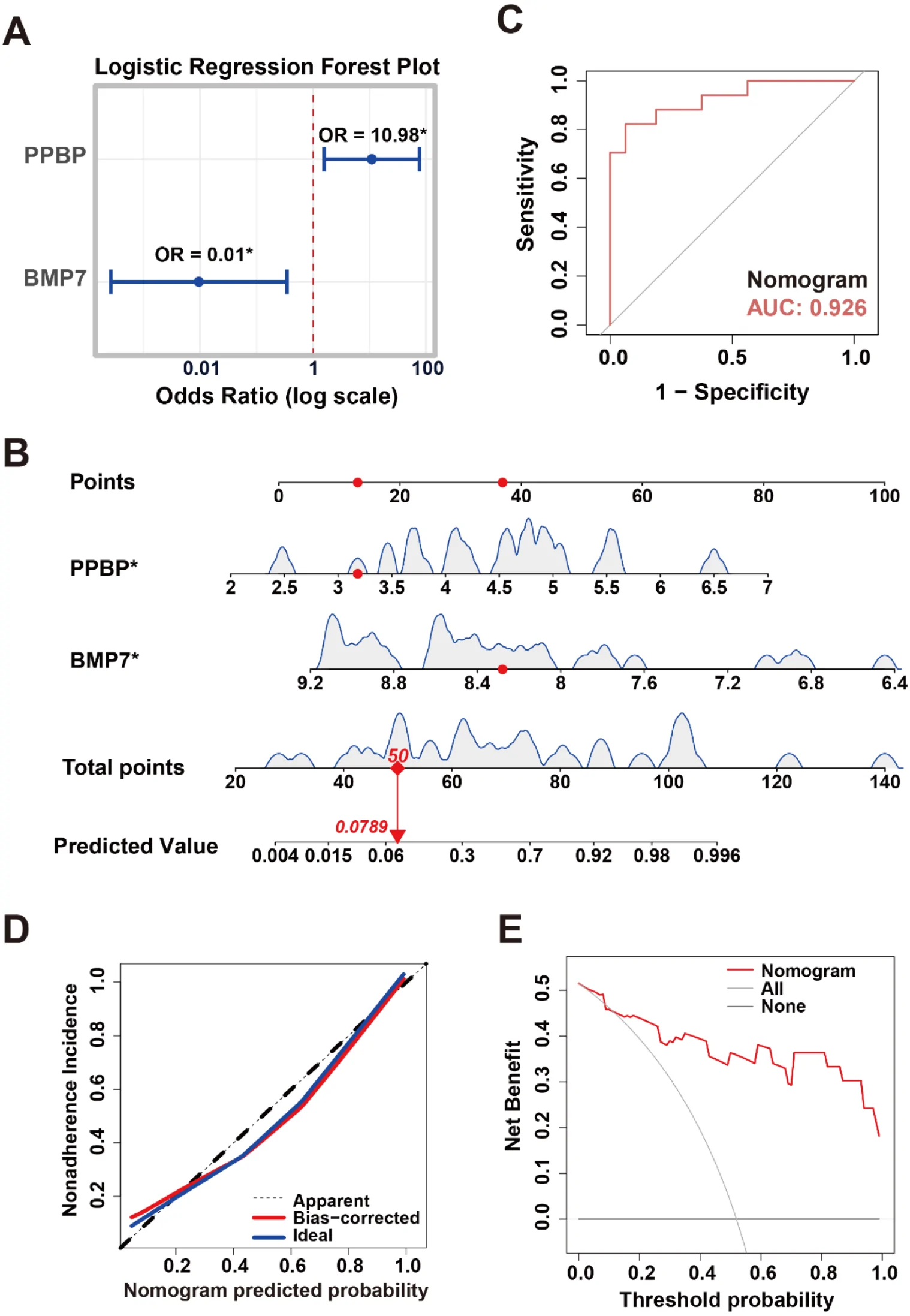
Diagnostic model in OSA-related AF based on two hub genes. **(A)** Logistic analysis; **(B)** Nomogram plot; **(C)** ROC analysis for nomogram; **(D,E)** Calibration curves and DCA analysis. OSA, Obstructive sleep apnea; AF, Atrial fibrillation.

Logistic analysis suggested that PPBP emerged as a significant predictor of OSA-related AF, with an OR of 10.98 (95% CI: 1.57–76.61, *P* = 0.016), whereas BMP7 showed a strong negative association with OSA-related AF (OR: 0.01, 95% CI: 0.00–0.35, *P* = 0.011). A forest plot was used to illustrate the association of PPBP and BMP7 with the risk of OSA-related AF (Figure 8A). To enhance diagnostic and predictive utility, a nomogram was constructed based on PPBP and BMP7 using logistic regression (Figure 8B). Receiver operating characteristic (ROC) analysis was performed to evaluate the diagnostic performance of the nomogram, with area under the curve (AUC) values used to assess sensitivity and specificity. The nomogram achieved AUC values exceeding 0.9, indicating strong diagnostic potential for OSA-related AF (Figure 8C). Calibration curves further demonstrated close alignment between predicted probabilities from the nomogram and ideal model outcomes (Figure 8D). Additionally, DCA supported the clinical utility of the nomogram, suggesting that its use in decision-making could improve diagnostic benefit for OSA-related AF (Figure 8E).

### 3.7 Immune infiltration

**FIGURE 9:**
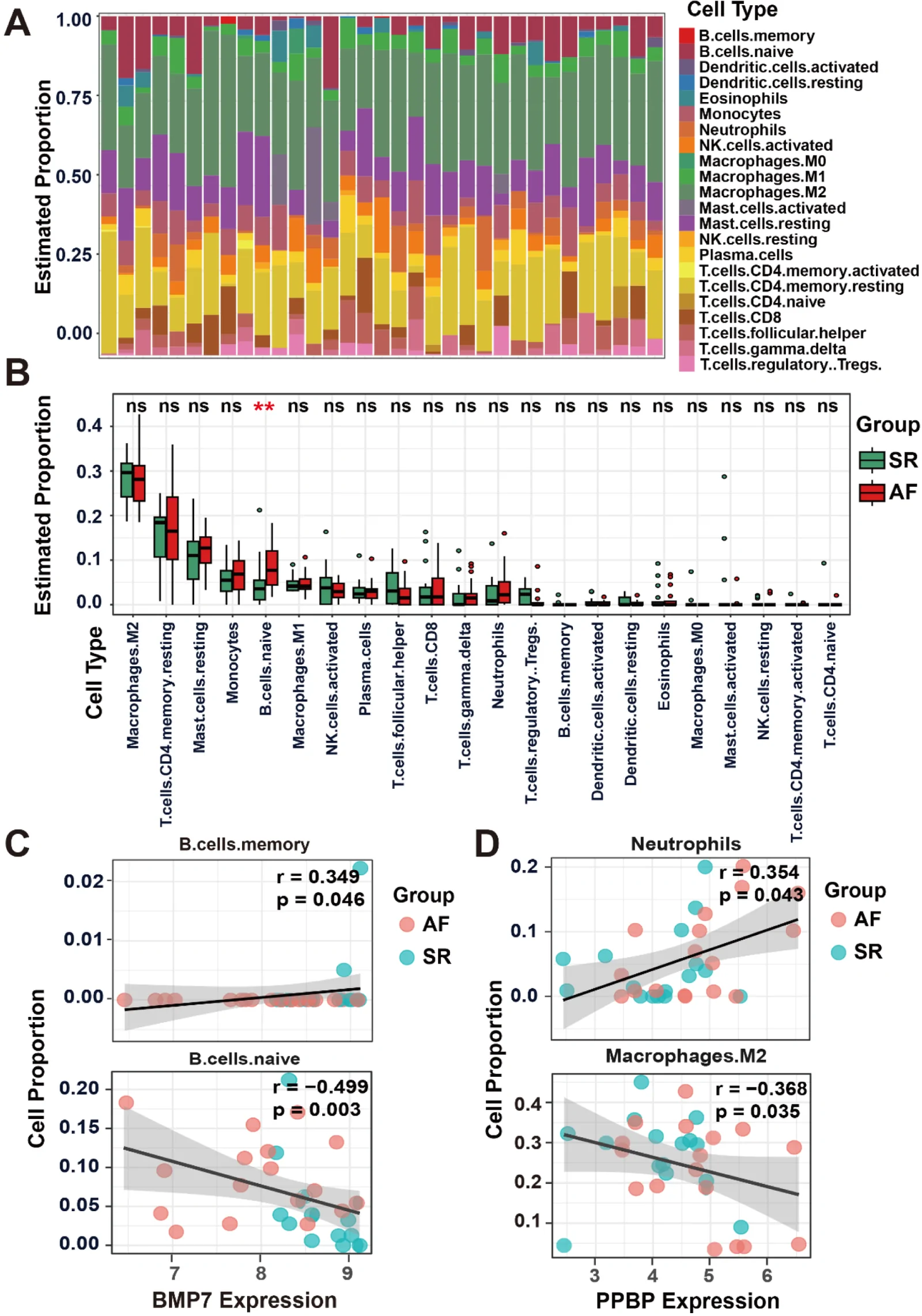
Immune infiltration. **(A)** The relative proportions of 22 immune cell types; **(B)** Immune proportion between control and AF samples, **represents *P* < 0.01; **(C,D)** Spearman correlation analysis between immune cell proportion and BMP7 expression and PPBP expression, respectively. AF, Atrial fibrillation.

Our enrichment analysis revealed that functional and pathway enrichment of OSA-related AF pathogenic genes was strongly linked to inflammatory and immune-related processes. To characterize immune cell infiltration and investigate its regulatory role in AF, the CIBERSORT algorithm was applied. This allowed us to assess immune cell composition and examine correlations between diagnostic biomarkers and specific immune cell subsets. Figure 9A illustrates the relative proportions of 22 immune cell types across individual samples. Compared to control samples, AF samples exhibited a notably higher proportion of naive B Cells (Figure 9B). Furthermore, the relationship between the expression levels of the two hub genes (PPBP and BMP7) and differentially abundant immune cell types was analyzed. As shown in Figures 9C,D, both PPBP and BMP7 displayed significant correlations with the infiltration levels of specific immune cell populations in AF.

### 3.8 Clinical cohort validation of the expression level of two hub genes and the evaluation of the diagnostic value of the nomogram models

**FIGURE 10:**
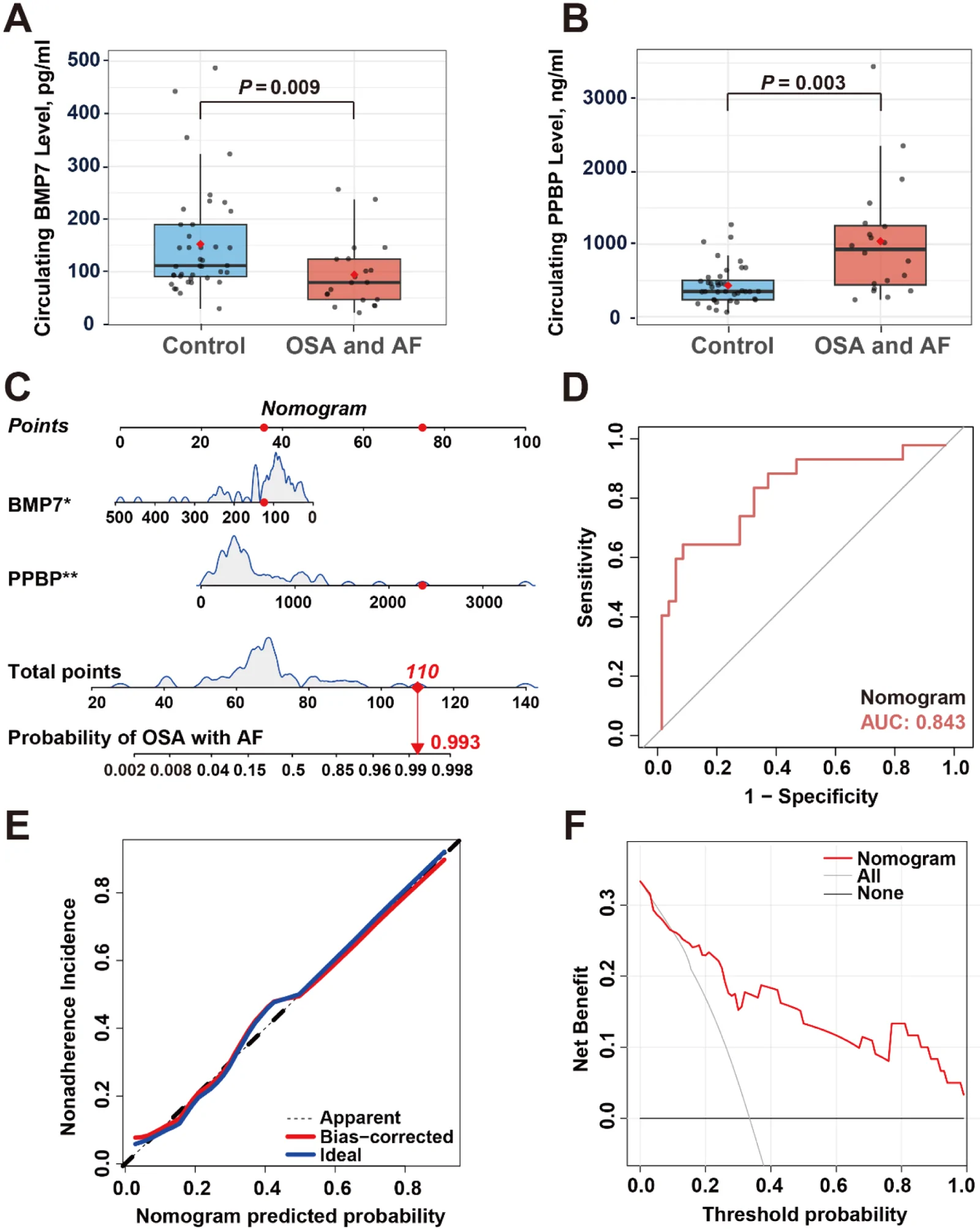
Construction and diagnostic evaluation of nomogram model based on BMP7 and PPBP for patients with clinical OSA-related AF. **(A)** Box plot showing the distribution of serum BMP7 levels in the two groups, with significantly lower levels observed in the OSA-related AF group compared to the control group (*P* = 0.009); **(B)** Box plot showing the distribution of serum PPBP levels in the two groups, with significantly higher levels observed in the OSA-related AF group compared to the control group (*P* = 0.003); **(C)** Nomogram constructed based on BMP7 and PPBP levels for the prediction of clinical OSA-related AF; **(D)** ROC curve of the nomogram (AUC: 0.843); **(E)** Calibration curve of the nomogram model for predicting clinical OSA-related AF; **(F)** DCA for the nomogram model. OSA: Obstructive sleep apnea; AF, Atrial fibrillation; ROC, Receiver operating characteristic; AUC, Area under curve; DCA, Decision curve analysis.

To validate the findings from the integrated bioinformatics analysis, we first assessed the expression levels of the two hub genes in a clinical cohort. Serum concentrations of PPBP and BMP7 were measured using ELISA. Results indicated significantly elevated PPBP levels and reduced BMP7 levels in patients with OSA and AF compared to control (Figures 10A,B). Moreover, we performed multivariable logistic regression analyses adjusting for BMI to evaluate the potential confounding effect of BMI. After adjustment, PPBP remained a significant predictor of OSA-related AF (adjusted OR = 1.35 per 100-unit increase, 95% CI: 1.14–1.67, *P* = 0.002), while BMP7 remained significantly protective (adjusted OR = 0.87 per 10-unit increase, 95% CI: 0.76–0.96, *P* = 0.019) (Supplementary Table S2), indicating that the diagnostic value of these two biomarkers is independent of BMI.

Subsequently, a diagnostic nomogram was developed based on this cohort to estimate the probability of OSA with AF (Figure 10C). ROC curve analysis demonstrated that the nomogram achieved a high AUC of 0.843 in distinguishing OSA with AF patients from control patients (Figure 10D). Furthermore, calibration curves and DCA supported the clinical utility of the nomogram, indicating that its use could improve predictive accuracy for OSA with AF (Figures 10E,F).

### 3.9 OSA rat model construction and related phenotypes verification

**FIGURE 11:**
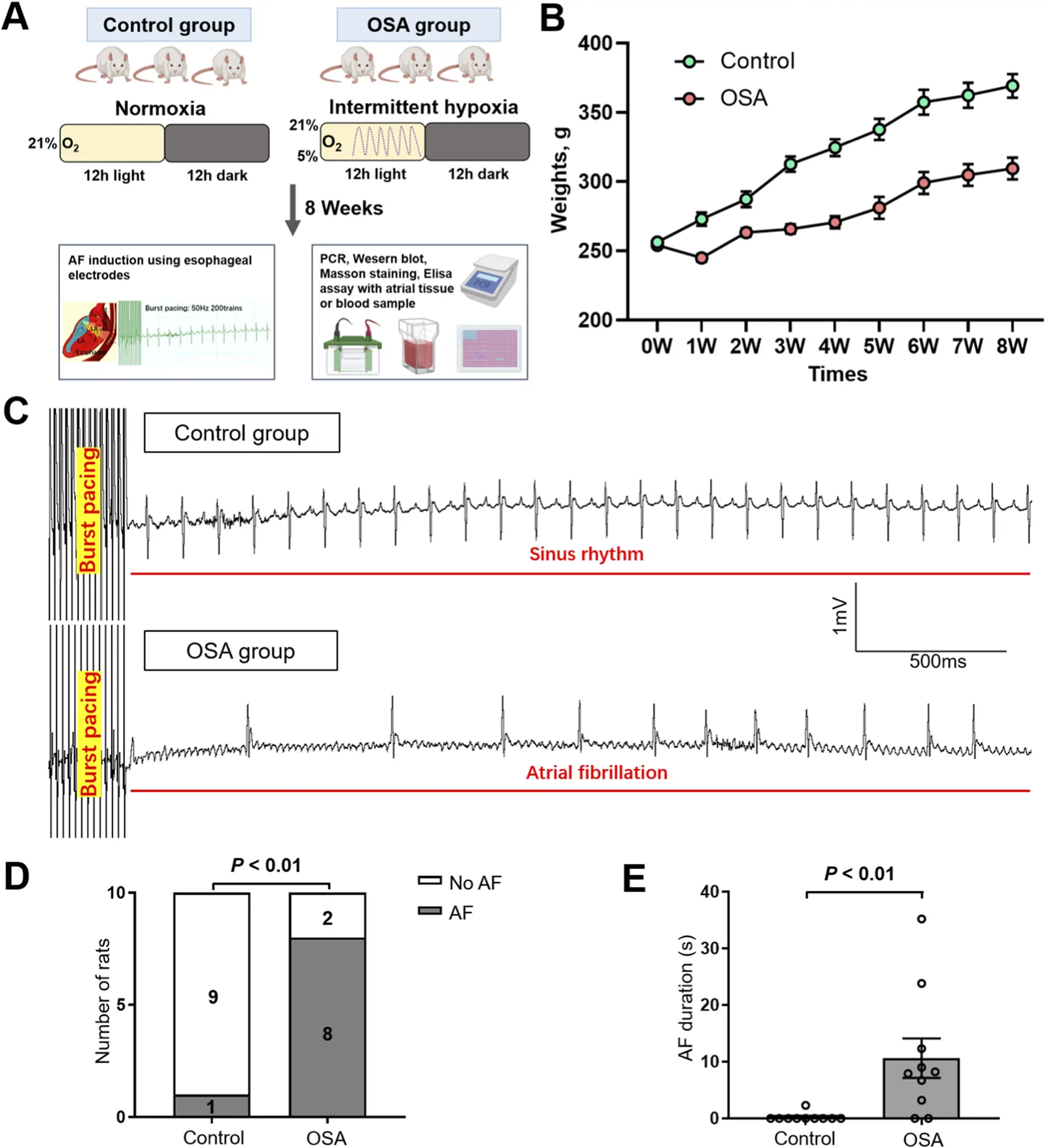
OSA rat model construction and AF induction. **(A)** Schematic representation for OSA rat model construction; **(B)** Weight recording between control and OSA rats; **(C)** AF induction between control and OSA group; **(D,E)** The statistical charts of AF inducibility and AF duration. OSA, Obstructive sleep apnea; AF, Atrial fibrillation.

**FIGURE 12:**
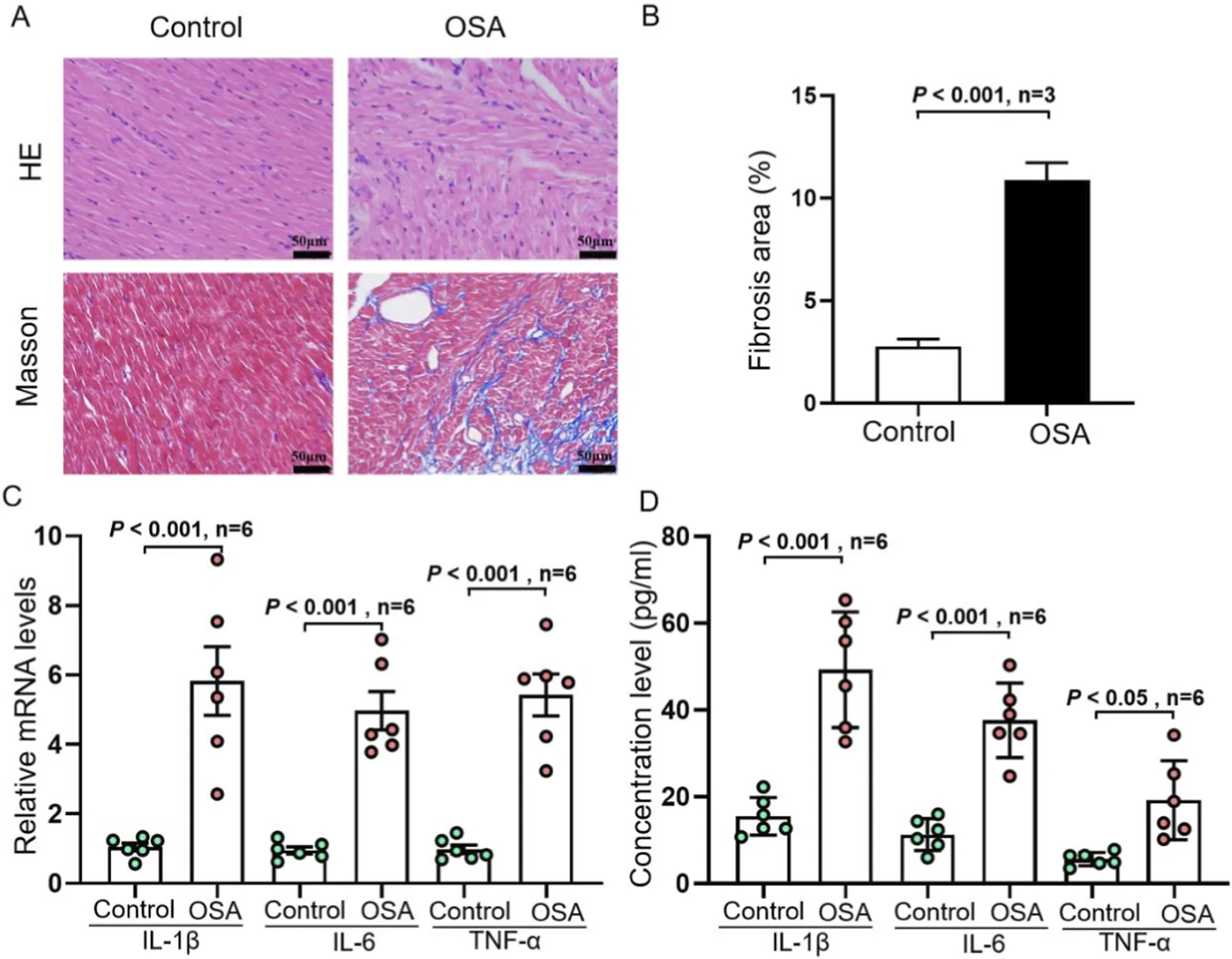
Histological and inflammatory factors detection. **(A)** HE and Masson staining for atrial tissue between control and OSA rats; **(B)** Statistical chart of fibrosis area (%) for Masson staining; **(C,D)** The mRNA and protein levels of inflammation-related factors (IL-1β, IL-6, and TNF-α) in atrial tissue between OSA and control rats, showing higher expression in the OSA group. OSA, Obstructive sleep apnea.

OSA rat model was successfully constructed (Figure 11A). Similar to previous study (Breuillard et al., 2023), body weight was lower in OSA rats than in control rats throughout the exposure (Figure 11B). Compared with control group, the AF induction probability was significantly higher in OSA group (OSA: 80% vs. control: 10%) (Figures 11C,D). Moreover, AF duration was longer in OSA group in comparison with control group (Figure 11E). Moreover, HE staining showed that the morphological structure of atrial tissue in the OSA rats was significantly disorganized compared with control rats. Masson staining also showed greater deposition of collagen fibers in the atrial tissues of OSA rats than in control rats (Figures 12A,B). Importantly, we detected the mRNA and protein levels of inflammation-related factors (IL-1β, IL-6, and TNF-α) in the atrial tissue between OSA rats and control rats, suggesting that inflammatory infiltration was more pronounced in the OSA group (Figures 12C,D).

### 3.10 Animal model validation of two hub genes

**FIGURE 13:**
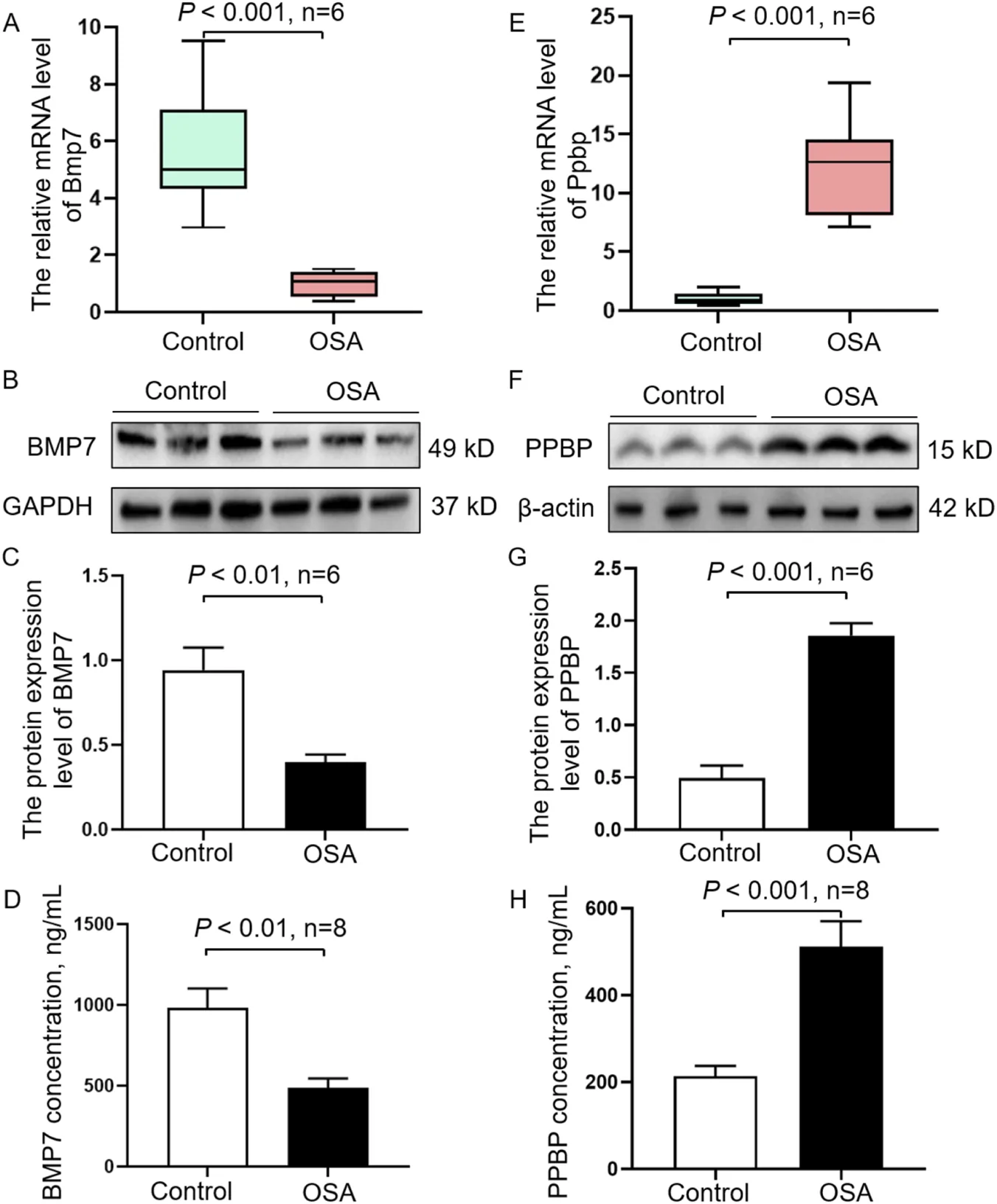
Two hub genes validation. **(A–C)** The mRNA and protein levels of BMP7 between control and OSA groups; **(D)** BMP7 concentration between control and OSA groups; **(E–G)** The mRNA and protein levels of PPBP between control and OSA groups; **(H)** PPBP concentration between control and OSA groups. OSA: Obstructive sleep apnea.

The mRNA level and protein level of hub genes were validated based on rat atrial tissue. Compared with control group, the BMP7 mRNA level was significantly lower and the PPBP mRNA level was significantly higher in OSA group, respectively (Figures 13A,E). Similarly, Western blot showed consistent results, suggesting that a lower BMP7 protein level and a higher PPBP protein level in OSA group (Figures 13B,C,F,G). Moreover, compared with control group, the blood BMP7 and PPBP concentrations were significantly lower and higher, respectively, in OSA group (Figures 13D,H). These results were consistent with the integrated bioinformatics analysis.

## 4 Discussion

In this study, we systematically integrate multiple levels analysis, including bioinformatics analysis, clinical cohort analysis, and animal model analysis, to identify the key genes and pathophysiological changes underlying OSA-related AF. The main findings are as follows: 1) Downregulated BMP7 and upregulated PPBP were screened as the key genes in OSA-related AF; 2) The activation of immune and inflammation might play a key role in the OSA-related AF.

Accumulating studies suggest OSA has a strong association with AF. The recent studies recommend the comorbidity and risk factor management, including OSA, served as the primary consideration for patient-centered AF therapy (Van Gelder et al., 2024; Chandan et al., 2025). Multiple evidence indicates that high prevalence of OSA in AF patients aggravate the maintenance and progression of AF. Moreover, OSA patients had been demonstrated a significantly increased risk of AF recurrence post-ablation therapy compared to patients without OSA (RR = 1.67, *P* < 0.05) (Fan et al., 2025). In addition, our previous meta-analysis study suggested that CPAP therapy could be an effective strategy to reduce AF recurrence risk by 42% post-ablation for AF with OSA patients (Li et al., 2023b). Whereas, OSA comorbid with AF still posed challenges due to the complicated and obscure molecular mechanisms. Therefore, further exploration is urgently needed through various level methods, such as bioinformatic analysis, animal model construction, as well as clinical cohort studies, to improve the prognosis of AF patients.

Similar to previous study reported (Zhu et al., 2023; Hu et al., 2024), our study focuses on secretory protein encoding genes based on their fundamental role as mediators of inter-organ communication. Although OSA and AF arise in distinct anatomical sites, their strong clinical association suggests the existence of systemic signaling pathways that might connect upper-airway dysfunction, metabolic dysregulation, and cardiac electrophysiological remodeling. Secretory proteins co-expressed between OSA and AF served as ideal molecular candidates for such remote crosstalk, as they can travel from source tissues to distal targets via the bloodstream. Our integrated multi-tissue analysis strategically leverages this paradigm. OSA-related adipose tissue and peripheral blood mononuclear cell datasets represent key sources of secretory factors, while the AF atrial tissue dataset represents the target organ where these circulating mediators may promote structural and electrical remodeling. By prioritizing genes encoding proteins likely to be secreted under OSA-related perturbations and capable of influencing atrial biology, we aimed to identify soluble mediators that directly link systemic OSA pathophysiology to local atrial substrate modification. This approach not only provides mechanistic insight into OSA and AF comorbidity but also highlights candidate circulating biomarkers and druggable targets for future translational investigation.

The progression from OSA to AF is driven by a network of interconnected biological pathways, centered on the systemic consequences of intermittent hypoxia and sleep fragmentation (Pintilie et al., 2025; Hendriks et al., 2021; Zhang et al., 2025). The cyclical pattern of hypoxia/normoxia alternation directly induces oxidative stress and activates inflammatory cascades, leading to systemic inflammation, which is a key mediator of atrial structural and electrical remodeling. Concurrently, sympatho-vagal imbalance and chemoreflex sensitization promote autonomic instability, lowering the threshold for atrial arrhythmogenesis (Deshmukh et al., 2025). Moreover, adipose tissue pathological changes, often associated with OSA, exacerbates this pathophysiology through adipokine dysregulation and enhanced pro-inflammatory signaling, which further amplifies endothelial dysfunction and promotes a pro-fibrotic atrial substrate (Ueland et al., 2025; Rafaqat et al., 2024b). Emerging evidence also suggests a role for gut dysbiosis in modulating systemic inflammation and metabolic homeostasis, potentially forming an additional pathway linking OSA to AF (Ren et al., 2026; Wu et al., 2024).

In our study, multiple biological functions analyses for OSA-related AF pathogenic genes were explored. The GO and KEGG analysis suggested that immune and inflammation activation played a significant role underlying OSA-related AF, which is specifically reflected in biology process of immune and inflammatory response, chemokine and cytokine activity of molecular function, as well as chemokine signaling pathway and NF-κB signaling pathway. Meanwhile, GSEA results indicated that multiple signaling pathway related to immune and inflammation were activated, such as chemokine signaling pathway and leukocyte trans-endothelial migration. Immune infiltration also showed immune dysfunction. Also, cMAP results showed that the action mechanism of potential pharmacological therapeutic agents was directly or indirectly related to the inhibition of inflammation and immune responses, such as PPAR receptor antagonist, PI3K inhibitor, and GSK inhibitor, which were expected to provide a solid theoretical basis for drugs involved in the treatment of OSA-related AF.

In addition, we successfully constructed OSA rat model as previous reported, and found that the AF induction rate was significantly elevated in the OSA group compared with the control group. Histological level study revealed that OSA rats showed a disorganized morphological structure and a more collagen fiber deposition of atrial tissue. These structural remodeling processes may increase susceptibility to arrhythmias and concurrently promote the formation of reentrant circuits (Frontera et al., 2026). To validate the biological functions results derived from bioinformation analysis, we detected the inflammatory factors levels (such as IL-1β, IL-6, and TNF-α) in the atrial tissues of OSA rats at the gene transcription level and protein expression level. The results indicated the inflammatory activation status of the atrial tissues of OSA rats, suggesting that our enrichment analysis was robust.

BMP7 (bone morphogenetic protein 7) is a member of the TGF-β superfamily, which is reported as a potent anti-inflammatory factor. A central mechanism underlying its anti-inflammatory activity is the promotion of phenotypic switching from pro-inflammatory monocytes or M1-type macrophages toward the anti-inflammatory M2 phenotype. This shift is accompanied by a marked reduction in pro-inflammatory cytokines such as IL-6, TNF-α, and monocyte chemoattractant protein-1, along with enhanced secretion of anti-inflammatory mediators including IL-10 (Da Silva et al., 2025; Aluganti Narasimhulu and Singla, 2020). In the context of cardiac remodeling, exogenous BMP7 has been shown to counteract TGF-β1 driven signaling and its downstream effectors Smad-2 and Smad-3, thereby attenuating myocardial fibrosis and promoting functional recovery after acute myocardial infarction (Jin et al., 2018). While direct evidence linking BMP7 to OSA remains limited, its established role in mitigating chronic inflammatory states suggests a plausible therapeutic potential in counteracting the systemic inflammation induced by OSA. In our study, bioinformatic analysis revealed that BMP7 expression was significantly downregulated, which was validated with consistent results at the gene and protein levels of OSA rat model. Notably, circulating BMP7 protein levels were significantly reduced in both OSA-related AF patients and OSA rat model, suggesting its potential utility as a diagnostic or prognostic biomarker for OSA-related AF.

PPBP, also known as C-X-C motif chemokine ligand 7, is primarily released in its active form as neutrophil-activating peptide-2. It is produced mainly by activated platelets and exhibits pro-inflammatory and pro-angiogenic properties. Beyond platelets, PPBP can also be secreted by other cell types, including neutrophils, megakaryocytes, natural killer cells, and lymphocytes, highlighting its broad role in inflammatory responses (Wang and Ward, 2026). Reportedly, PPBP might be one of the central hub genes associated with cardiovascular diseases. During vascular injury, PPBP could recruit neutrophils and promotes angiogenesis via activation of the CXCR2 receptor. However, sustained neutrophil activation can exacerbate initial damage, contributing to thrombosis-related cardiovascular and inflammatory disorders (Horioka et al., 2019). In addition, CXCR2 signaling has been shown to promote monocyte mobilization, which plays a pathogenic role in AF development (Zhang et al., 2020). In addition, OSA induces intermittent hypoxia, leading to systemic inflammation and oxidative stress (Lv et al., 2023). PPBP is markedly expressed in lung fibroblasts and may contribute to pulmonary fibrosis. Moreover, elevated PPBP levels in lung tissue have been linked to severe COVID-19, suggesting its potential as a biomarker for respiratory disease severity (Wismans et al., 2023). Notably, our study suggested that PPBP expression was significantly upregulated based on bioinformatic analysis and tissue expression analysis of OSA rat model, as well as circulating PPBP protein detection using the blood sample of OSA-related AF patients and OSA rat model, suggesting that our results were robust. Moreover, immune infiltration results showed that PPBP expression was positively correlated with neutrophils proportion and negatively correlated with macrophages M2 proportion, suggesting that PPBP might play a pro-inflammatory role on the pathophysiology of OSA-related AF.

Importantly, to address potential confounding by BMI, we performed multivariable logistic regression analyses adjusting for BMI in addition to other baseline covariates. The associations of PPBP and BMP7 with OSA-related AF remained statistically significant after adjustment, suggesting that their diagnostic value is independent of BMI. This finding is particularly meaningful given that recent study has shown that the causal effect of OSA on AF is attenuated after BMI adjustment (Li Y. et al., 2023), highlighting the need to identify BMI-independent mechanistic pathways. Our results suggest that BMP7 and PPBP may represent such pathways, offering potential biomarkers and therapeutic targets that are not merely proxies for obesity. However, we acknowledge that the cross-sectional nature of the clinical cohort limits causal inference, and the observed BMI difference between groups reflects the known epidemiological link between obesity and OSA.

Despite the encouraging results in our study, several limitations should be emphasized. First, the datasets analyzed in this study were retrieved from the GEO database without independent external database validation, and the relatively modest sample sizes may increase the risk of false-positive findings. Additionally, recent single-cell studies have preliminarily revealed T Cell alterations in AF (Long et al., 2025), highlighting the need for future investigations at the single-cell resolution to further elucidate the pathogenesis of OSA-associated AF. Validation in larger, independent cohorts and deeper single-cell profiling will be essential to confirm our findings and uncover the cellular heterogeneity underlying this comorbidity. Second, our study focused on the screening and validation of pathological phenomena of OSA-related AF, without exploring mechanisms via gene knockout or inhibitor studies. This also represents a key area of focus for our team in the future. Third, the OSA rat model utilized in this study is based on intermittent hypoxia induction. Notably, we observed weight loss in the OSA group, which appears inconsistent with the common clinical phenotype of obesity-induced OSA. This phenomenon is likely associated with intermittent hypoxia induced hypermetabolism, elevated energy expenditure, and systemic stress responses (Olea et al., 1985; Gagnon et al., 2023). Consequently, this metabolic discrepancy may impact the interpretation of BMP7 and PPBP expression when translating our findings to obese human patients. Therefore, while this specific modeling approach has been previously reported and validated for investigating OSA-related pathological mechanisms (Breuillard et al., 2023), caution is warranted in extrapolating these results to the broader OSA population, and future studies combining intermittent hypoxia with obesity models are needed to further validate our findings. Fourth, we acknowledge that the limited number of overlapping genes is partly due to our secretome-focused screening strategy and stringent thresholds. This may limit the scope of our findings, although we do not interpret this as evidence against OSA-AF comorbidity. Larger-scale studies are needed to further validate our results. Finally, while LVEF was comparable between groups, we acknowledge the lack of detailed diastolic function assessment (e.g., E/e' ratio, left atrial volume index) in our clinical cohort. Such parameters would provide more sensitive measures of OSA-related cardiac dysfunction in HFpEF patients and should be incorporated in future studies.

## 5 Conclusion

Based on the findings of this study, the downregulated BMP7 and upregulated PPBP may serve as key molecular features in the pathogenesis of OSA-related AF, potentially operating through pathways independent of obesity-related confounders. These results also highlight a shared pathological pathway between OSA and AF, providing novel perspectives for the diagnosis of this comorbidity and supporting the potential development of serum-based biomarkers and targeted therapeutic strategies.

## Data Availability

The datasets presented in this study can be found in online repositories. The names of the repository/repositories and accession number(s) can be found in the article/Supplementary Material.
