# Correction: Unveiling the inflammatory link: integrated analysis identifies BMP7 and PPBP as key genes in obstructive sleep apnea-related atrial fibrillation

**DOI:** 10.3389/fcell.2026.1988944

**Published:** 2026-09-17

**Authors:** Feng Li, Xing Xu, Yi-Qiu Hu, Cheng-Peng Zhang, Yi-Wei Shen, Chi Geng, Jia-Peng Chu, Hui-Yu Bai, Xiao-Song Gu, Hui Li

**Affiliations:** Department of Cardiology, The Second Affiliated Hospital of Soochow University, Suzhou, Jiangsu, China

**Keywords:** atrial fibrillation, obstructive sleep apnea, secretory proteins, immune cell infiltration, clinical cohort, rat model

There was an error in Figure 11D. The bar representing “Control” was placed on the right and the bar representing “OSA” was placed on the left, which was the reverse of their labels. The corrected Figure 11 appears below.

**FIGURE 11:**
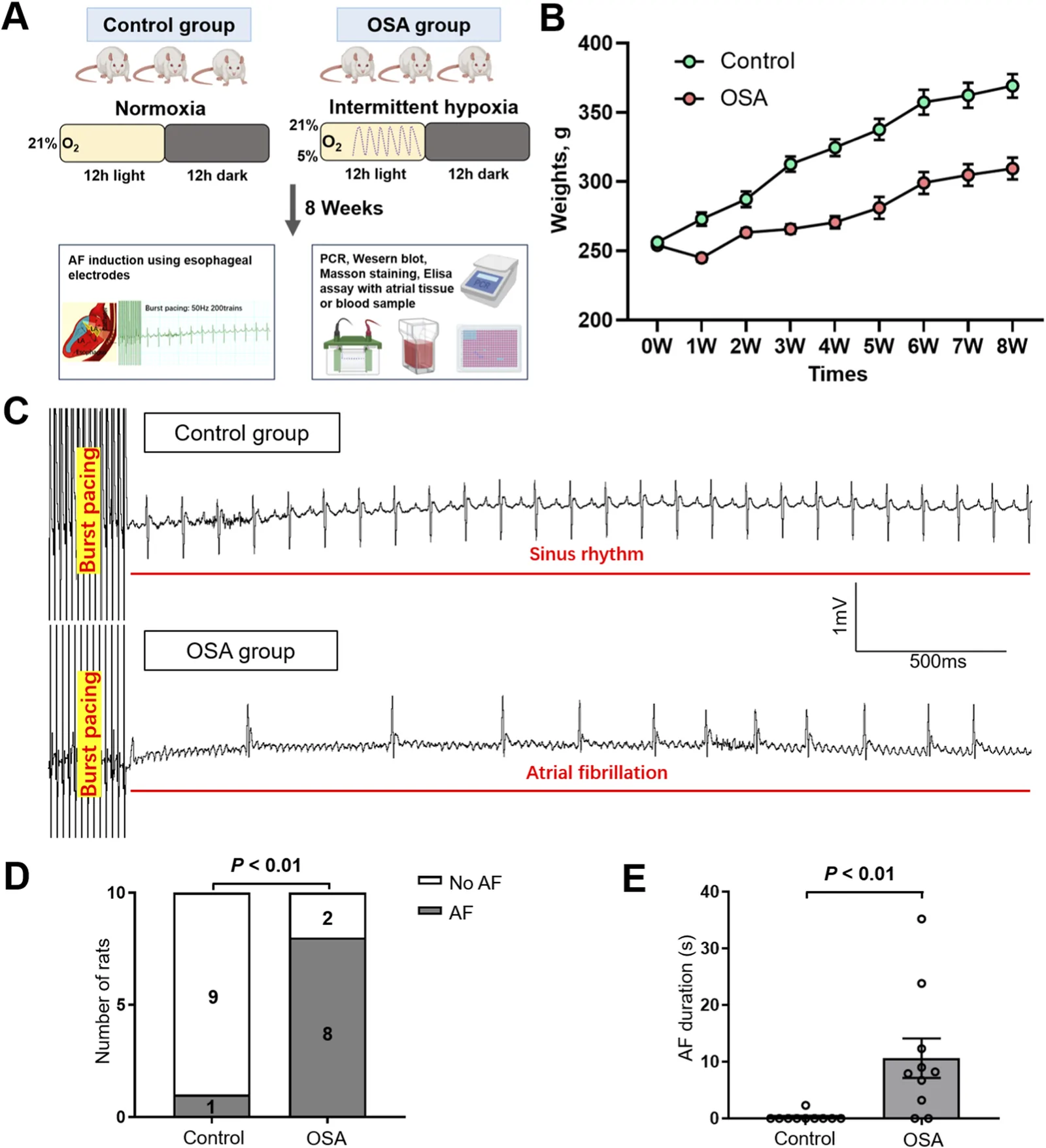
OSA rat model construction and AF induction. **(A)** Schematic representation for OSA rat model construction; **(B)** Weight recording between control and OSA rats; **(C)** AF induction between control and OSA group; **(D,E)** The statistical charts of AF inducibility and AF duration. OSA, Obstructive sleep apnea; AF, Atrial fibrillation.

The original version of this article has been updated.

