# Supplementary material for: Unveiling the inflammatory link: integrated analysis identifies BMP7 and PPBP as key genes in obstructive sleep apnea-related atrial fibrillation
Source: Front Cell Dev Biol. 2026 Aug 20;14:1914540. doi: 10.3389/fcell.2026.1914540 (PMC13538095; doi:10.3389/fcell.2026.1914540)
Supplement: Supplementary file 1 [file Supplementaryfile6.pdf]

Supplementary Methods

Machine learning

To identify hub genes for constructing an AF diagnostic model, a two-stage feature selection strategy was applied. First, LASSO regression was performed using the ‘glmnet’ package (version 4.1.10) with 10-fold cross-validation based on Binomial Deviance. The optimal regularization parameter λ was selected at λ_{1se}, corresponding to the most regularized model within one standard error of the minimum deviance, to obtain a sparse set of non-zero coefficient genes. Second, the random forest algorithm was implemented using the ‘randomForest’ package (version 4.7.1.2) with ntree = 1000 and mtry = 6, and gene importance was evaluated by MeanDecreaseGini over 100 independent runs. Genes with a MeanDecreaseGini score > 2 were retained. The final hub genes were defined as the intersection of genes selected by both methods (non-zero coefficients at λ_{1se} and MeanDecreaseGini > 2)[1]. These genes were subsequently utilized to develop a diagnostic model for OSA-related AF. To confirm that the selected genes encode experimentally validated secretory proteins, hub genes were further screened using the GeneCards database (https://www.genecards.org/, version 5.25) based on the presence of experimental evidence for "Secreted" classification in their subcellular localization annotation.

Reference

[1] Motamedi F, Pérez-Sánchez H, Mehridehnavi A, et al. Accelerating big data analysis through LASSO-Random forest algorithm in QSAR studies. Bioinformatics. 2022. 38(2): 469-475.
