# Supplementary material for: Unveiling the inflammatory link: integrated analysis identifies BMP7 and PPBP as key genes in obstructive sleep apnea-related atrial fibrillation
Source: Front Cell Dev Biol. 2026 Aug 20;14:1914540. doi: 10.3389/fcell.2026.1914540 (PMC13538095; doi:10.3389/fcell.2026.1914540)
Supplement: Supplementary file 2 [file Supplementaryfile3.docx]

**Supplementary Table 2.** Association of PPBP and BMP7 with OSA-related AF before and after adjustment for BMI.

| Variable | Univariate logistic regression | | | Multivariate logistic regression (Adjusted BMI) | | |
| --- | --- | --- | --- | --- | --- | --- |
|  | OR | 95% CI | *P* | OR | 95%CI | *P* |
| PPBP (per 100-unit increase) | 1.34 | (1.14 - 1.64) | 0.001 | 1.35 | (1.14 - 1.67) | 0.002 |
| BMP7 (per 10-unit increase) | 0.90 | (0.80 - 0.98) | 0.035 | 0.87 | (0.76 - 0.96) | 0.019 |

**Abbreviation:** OSA: Obstructive sleep apnea; AF: Atrial fibrillation; BMI: Body mass index; OR: Odds ratio; 95% CI: 95% confidence interval
