# Supplementary material for: Unveiling the inflammatory link: integrated analysis identifies BMP7 and PPBP as key genes in obstructive sleep apnea-related atrial fibrillation
Source: Front Cell Dev Biol. 2026 Aug 20;14:1914540. doi: 10.3389/fcell.2026.1914540 (PMC13538095; doi:10.3389/fcell.2026.1914540)
Supplement: Supplementary file 3 [file Supplementaryfile4.docx]

**Supplementary Figure 1**

**Supplementary Figure 1. Screening the key genes for the OSA-related AF.** A. Veen plot with overlapping genes between OSA-associated secretory proteins and AF-related key genes; B-C. LASSO regression analysis based on six key genes; D-E. RF algorithm; OSA: Obstructive sleep apnea; AF: Atrial fibrillation; RF: Random Forest.
