# Supplementary material for: Unveiling the inflammatory link: integrated analysis identifies BMP7 and PPBP as key genes in obstructive sleep apnea-related atrial fibrillation
Source: Front Cell Dev Biol. 2026 Aug 20;14:1914540. doi: 10.3389/fcell.2026.1914540 (PMC13538095; doi:10.3389/fcell.2026.1914540)
Supplement: Supplementary file 4 [file Supplementaryfile5.docx]

**Supplementary Figure 2**

**Supplementary Figure 2. Expression analysis for four key genes based on the AF dataset and the OSA-related adipose tissue dataset**. OSA: Obstructive sleep apnea; AF: Atrial fibrillation. OSA: Obstructive sleep apnea; AF: Atrial fibrillation
